# Transposase-CRISPR mediated targeted integration (TransCRISTI) in the human genome

**DOI:** 10.1038/s41598-022-07158-8

**Published:** 2022-03-01

**Authors:** Mahere Rezazade Bazaz, Mohammad M. Ghahramani Seno, Hesam Dehghani

**Affiliations:** 1 Division of Biotechnology, Faculty of Veterinary Medicine, Ferdowsi University of Mashhad, 9177948974 Mashhad, Iran; 2 Stem Cell Biology and Regenerative Medicine Research Group, Research Institute of Biotechnology, Ferdowsi University of Mashhad, Azadi Square, 9177948974 Mashhad, Iran; 3 Department of Basic Sciences, Faculty of Veterinary Medicine, Ferdowsi University of Mashhad, 9177948974 Mashhad, Iran

**Keywords:** Gene delivery, Genetic techniques, Molecular medicine

## Abstract

Various methods have been used in targeted gene knock-in applications. CRISPR-based knock-in strategies based on homology-independent repair pathways such as CRISPR HITI have been shown to possess the best efficiency for gene knock-in in mammalian cells. However, these methods suffer from the probability of plasmid backbone insertion at the target site. On the other hand, studies trying to combine the targeting ability of the Cas9 molecule and the excision/integration capacity of the PB transposase have shown random integrations. In this study, we introduce a new homology-independent knock-in strategy, Transposase-CRISPR mediated Targeted Integration (TransCRISTI), that exploits a fusion of Cas9 nuclease and a double mutant *piggyBac* transposase. In isogenic mammalian cell lines, we show that the TransCRISTI method demonstrates higher efficiency (72%) for site-specific insertions than the CRISPR HITI (44%) strategy. Application of the TransCRISTI method resulted in site-directed integration in 4.13% and 3.69% of the initially transfected population in the human AAVS1and PML loci, respectively, while the CRISPR HITI strategy resulted in site-directed integration in the PML locus in only 0.6% of cells. We also observed lower off-target and random insertions in the TransCRISTI group than the CRISPR HITI group. The TransCRISTI technology represents a great potential for the accurate and high-efficiency knock-in of the desired transposable elements into the predetermined genomic locations.

## Introduction

Targeted gene knock-in methods have been used in a wide range of applications such as gene therapy, gene correction, or the study of gene function^1^. Various approaches, such as those that use zinc-finger nucleases (ZFNs), TALENs (transcription activator-like effector nucleases), and CRISPR/Cas (clustered regularly interspaced short palindromic repeats/CRISPR-associated), have been applied to induce site-specific genome modifications in mammalian cells. Targeted integration of gene fragments into the genome using ZFNs and TALENs is complex and time-consuming. However, using the CRISPR/Cas system for targeted integration of gene fragments is a simple and easy approach that works based on the creation of DSB (double-strand DNA break) at the target site^2^. Targeted DNA breaks generated by the site-specific nuclease activity of the Cas endonuclease will trigger intracellular repair pathways which can be generally categorized as homology-dependent and homology-independent repair pathways^3–5^. Homology-dependent (HR) pathways allow low efficiency of gene knock-in, especially in non-dividing cells and in in vivo gene knock-in approaches.

On the other hand, the homology-independent repair pathways exploit the non-homologous end joining (NHEJ) throughout the cell cycle in dividing and non-dividing cells^6^. The homology-independent repair pathways show higher efficiency in joining genomic DNA fragments, and despite their error-prone nature^7^, have been employed in different CRISPR-based knock-in strategies such as HITI (homology-independent targeted insertion)^8^, and KiBL (knock-in blunt ligation)^9^. The CRISPR HITI strategy has been shown to possess the best efficiency for gene knock-in in mammalian cells. The donor in the HITI method is simple to produce and does not need the cloning of the flanking homology arms, synthesis of large ssDNA donor fragments, or any other modifications. In this strategy, the sgRNA binding site(s) are placed at one or either side of the gene of interest (GOI) cassette. These sites on the donor molecule when targeted by Cas9 (CRISPR-associated protein 9), provide a linear cassette that will be integrated into the genome. The HITI knock-in strategy is associated with certain drawbacks. One relates to the probability of plasmid backbone insertion at the target site, which would increase the size of the insert and elicit an immune response to the bacterial DNA fragments. Further, more cloning steps are required to devise sgRNA binding site(s) in the donor cassette.

Several studies have tried to combine the targeting ability of the Cas9 molecule and the excision/integration capacity of the PB transposase. The fusion of the catalytically dead Cas9 (dCas9) to PB transposase was unsuccessful in site-directed integrations of the HPRT locus^10^. Another study showed that the excision competent/integration defective (Exc^+^ Int^−^) hyperactive *piggyBac* transposase^11,12^ fused to dCas9 was able to target the CCR5 safe harbor sequence, albeit inefficiently^13^. We have recently shown that dCas9.PB using dual sgRNAs can achieve site-directed transposition in 0.32% of cells out of the initially transfected population^14^. In that study, we designed and used a promoter/reporter complementation assay to register cells with specific integrations, where only by complementation upon correct genomic integration, the reporter is activated. However, the strategy of dCas9.PB using dual sgRNAs similar to the previously published methods suffered from off-target and random integrations.

In this study, we introduce a new homology-independent knock-in strategy that relies on the excision capability of the *piggyBac* transposase (PB) and the site-specific integration ability of Cas9. This strategy, hereafter called TransCRISTI (Transposase-CRISPR mediated Targeted Integration), exploits an effector molecule (Cas9.PB^dm^) generated by fusing Cas9 to a double mutant (Excision^+^/Integration¯) piggyBac transposase. Here, the random integration activity of PB and its ability for re-integration of the transposon into another region is prevented by the introduced mutations. Since this mutated transposase is fused to the Cas9 nuclease, it can provide its tethered transposon fragment for Cas9-mediated integration. In this study, we use the promoter/reporter complementation assay to register cells with specific integrations, and show that TransCRISTI has higher efficiency for site-specific insertions and lower off-target and random insertions than the CRISPR HITI in mammalian cells. Using this strategy we were able to perform site-directed integration in the human AAVS1 safe harbor genomic region in 4.13%, and in the PML locus in 3.69% of the total transfected HEK293T cells, while application of CRISPR HITI resulted in site-directed integration in the PML locus in only 0.6% of total transfected cells. In isogenic cell lines, the TransCRISTI method demonstrated higher efficiency (72% of cell lines; *n* = 150) for site-specific insertions than the CRISPR HITI method (44% of cell lines; *n* = 150). The TransCRISTI technology presents great potentials for accurate and high-efficiency knock-in of DNA elements into desired genomic locations.

## Results

### The Cas9.PB^dm^ effector molecule performs a specific interplasmid knock-in

**Figure 1:**
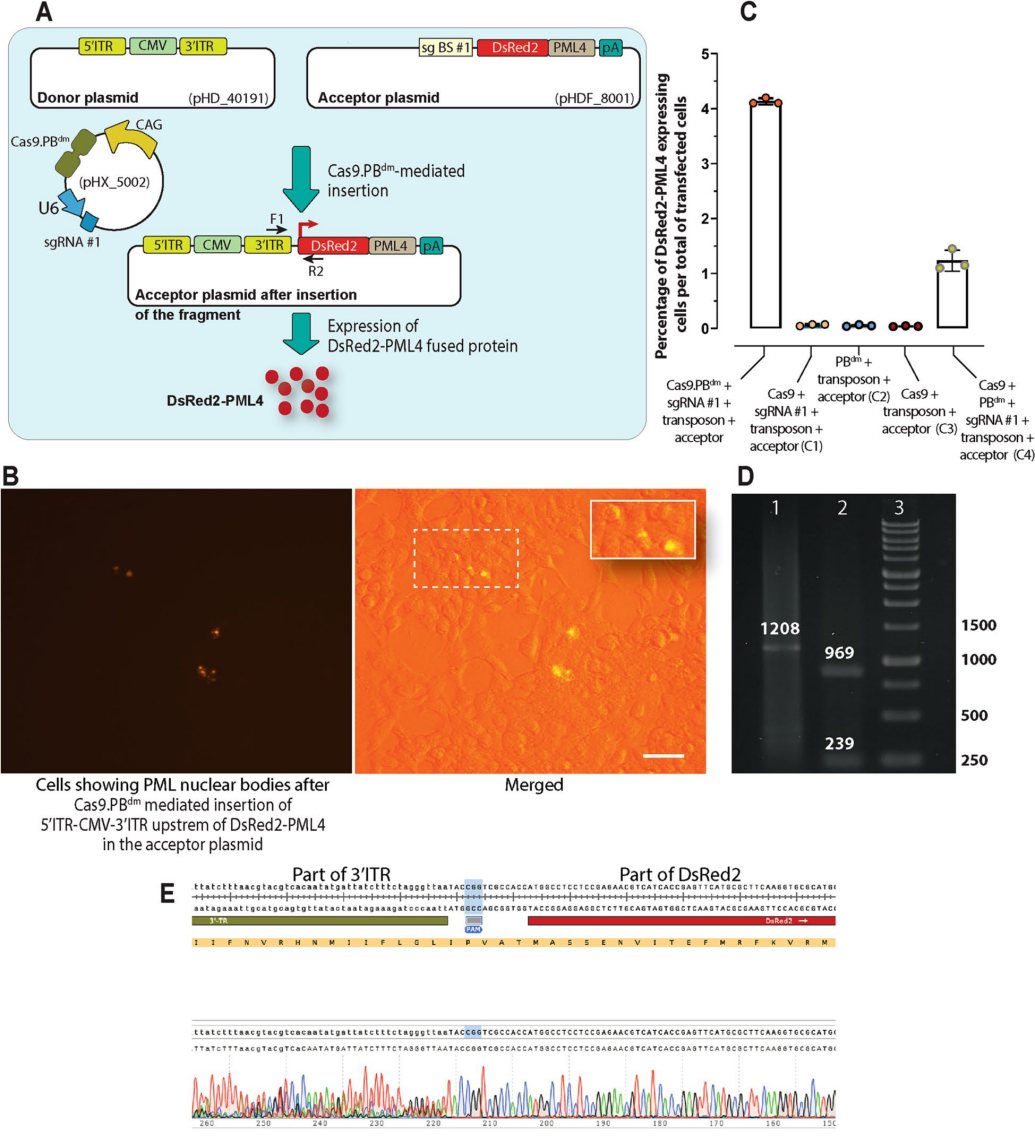
Interplasmid knock-in assay mediated by TransCRISTI. (**A**) Schematic illustration of the donor plasmid containing 5′ITR-CMV promoter-3′ITR (pHD_40191), the acceptor plasmid containing a binding site for sgRNA #1 (sg BS #1) upstream of a promoterless DsRed2-PML4-polyA signal (pA) (pHDF_8001), and a plasmid encoding Cas9.PB^dm^ effector molecule and sgRNA #1 (pHX_5002). A new plasmid is generated by the Cas9.PB^dm^-mediated integration of the donor transposon upstream of the promoterless reporter in HEK293T cells leading to the expression of punctate PML nuclear bodies with red fluorescence. (**B**) The left panel is the fluorescent image of DsRed2-PML4 expressing cells 48 h post-transfection. The DsRed2-PML4 expressing cells are considered as positive cells for the integration of the donor fragment (containing CMV promoter flanked by PB transposon ITRs) into the sgRNA #1 binding site (sg BS #1) upstream of the promoterless acceptor construct. In the right panel which is a merged brightfield and fluorescent image, the inset in the upper right corner shows an enlarged area of the image outlined by a dashed square containing several nuclei with red PML nuclear bodies. The scale bar indicates a length of 100 μm. (**C**) DsRed2-PML4 expressing cells were counted in each experimental group. Group 1 (TransCRISRI group) cells were transfected with plasmids encoding Cas9.PB^dm^ and sgRNA #1, and also the donor and acceptor plasmids. The plasmid components are shown for four control groups (C1, C2, C3, and C4). Results are represented as the percentages of the DsRed2-PML4 positive cells over the total transfected cells. The statistical differences between the TransCRISRI group and the control groups were analyzed by the Mann–Whitney U test. (**D**) Gel electrophoresis analysis of PCR amplicons to validate the integration events. PCR amplicon in lane 1 represents the recovery of the insertion site by F1 primer on the donor construct and R2 on the acceptor construct (Tables S1, S2) which results in the amplification of a 1208 bp PCR product. Lane 2 is a digestion product of the 1208 bp amplicon by the AgeI restriction enzyme resulting in two bands of 969 and 239 bps. Lane 3 is a 1 Kb DNA size marker. (**E**) Sanger sequencing analysis of the targeted region after Cas9.PB^dm^-mediated insertion of 5′ITR-CMV-3′ITR upstream of DsRed2 gene showing one electropherogram trace.

We first analyzed the site-specific knock-in ability of the Cas9.PB^dm^ effector, generated by the fusion of Cas9 and double mutant PB (PB^dm^), in an interplasmid knock-in assay. The acceptor plasmid contained a sgRNA binding site (sg BS #1; Fig. 1A) upstream of a promoterless DsRed2-PML4 reporter. The donor plasmid contained a 5′ITR-CMV promoter-3′ITR *piggyBac* transposon. Co-transfection of the acceptor (pHDF_8001) and donor (pHD_40191) plasmids along with the plasmids encoding sgRNA #1 and Cas9.PB^dm^ (pHX_5002) in HEK293T cells resulted in the displacement of the fragment 5′ITR-CMV-3′ITR from the donor plasmid into the acceptor plasmid upstream of the reporter, leading to the expression of DsRed2-PML4 protein. The expressed DsRed2-PML4 was accumulated in PML nuclear bodies and generated distinct nuclear fluorescent punctations (Fig. 1B). To determine the exact site-specific integration events, the percentage of DsRed2-PML4 positive cells over the total live transfected cells was calculated for all experimental groups. The results showed that there were 3.35-fold higher interplasmid events in the fused effector group than those in the group with separate effectors (*p* < 0.05; 4.13% vs. 1.23% DsRed2-PML4 positive cells, respectively) (Fig. 1C). The percentage of DsRed2-PML4 positive cells in the C1 control group (having Cas9 instead of Cas9.PB^dm^), C2 control group (having PB^dm^ instead of Cas9.PB^dm^), and the C3 control group (having Cas9 in the absence of sgRNA) were 0.065%, 0.053%, and 0.041%, respectively. Hence, the interplasmid knock-in assay revealed that the sgRNA-guided Cas9.PB^dm^ effector has a higher ability for site-specific insertion of a transposable element in comparison with the separated Cas9 and PB^dm^ effectors. To verify the interplasmid displacement event between the donor and acceptor plasmids, the insertion region was amplified by PCR (Fig. 1D) and subjected to Sanger sequencing (Fig. 1E).

### TransCRISTI can be used for site-directed integration in a safe harbor region

**Figure 2:**
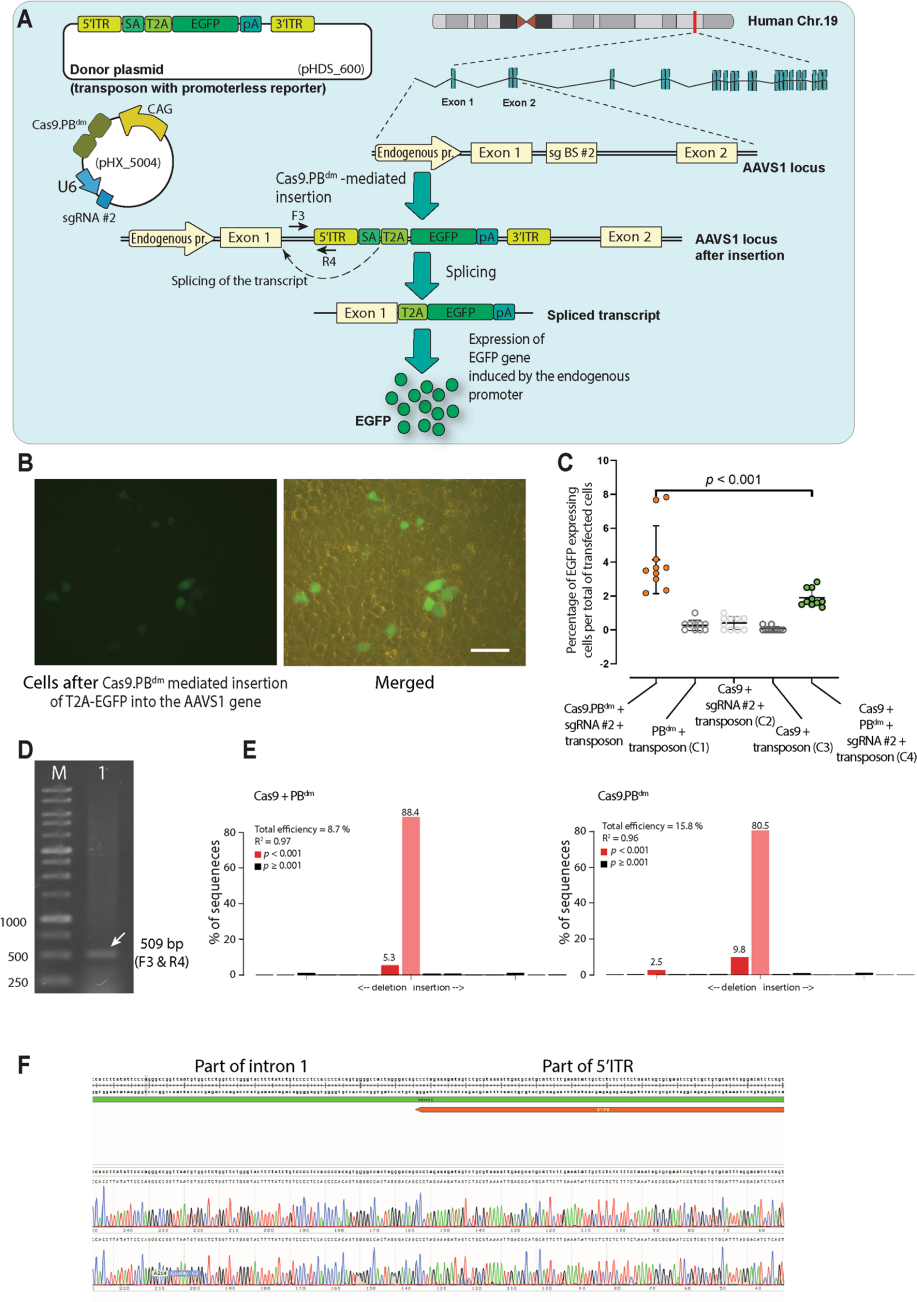
Intron-based gene knock-in mediated by TransCRISTI. (**A**) Schematic illustration of the gene-trap knock-in method by TransCRISTI in the human AAVS1 gene (ACNO. S51329). HEK293T cells were cotransfected with a plasmid encoding Cas9.PB^dm^ effector and sgRNA #2 (pHX_5004) and a gene-trap donor construct (pHDS_600) (Table S1). Donor plasmid contains a splice acceptor site (SA), a sequence encoding T2A self-cleaving peptide, EGFP gene, and polyA signal flanked by *piggyBac* 5′ITR and 3′ITR. The transposon fragment integrates into the sgRNA binding site #2 (sgBS #2) in the first intron of the AAVS1 gene. In the transcript produced under the control of endogenous AAVS1 promoter, the SA sequence promotes the attachment of the T2A to the exon1 of AAVS1 and splicing out of 5′ITR, leading to a mature transcript that can be translated to EGFP protein. (**B**) The left panel is the fluorescent image of EGFP expressing cells, 48 h post-transfection. The EGFP expressing cells were considered positive for the intron-based insertion of the promoterless construct into the sgBS #2 sequence (left panel). The right panel is a merged image of brightfield and fluorescent. The scale bar indicates a length of 100 μm. (**C**) EGFP-expressing cells were counted in each experimental group. Group 1 (TransCRISRTI group) cells were transfected with plasmids encoding Cas9.PB^dm^, sgRNA #2, and a plasmid containing the transposon. The plasmid components are shown for four control groups (C1, C2, C3, and C4). Results are represented as the percentages of the EGFP positive cells over the total transfected cells. The statistical differences between the TransCRISRTI group and the control groups were analyzed by the Mann–Whitney U test. *p < 0.001. (**D**) Gel electrophoresis of PCR fragments to confirm the specific knock-in event at the first intron of the AAVS1 locus. F3 primer located on the first intron of AAVS1 and R4 primer on 5′ITR in the donor construct (Tables S1, S2) were used to amplify a 509 bp product (lane 1) in the PCR reaction of genomic DNA of cells with specific insertion. Lane M is a 1 Kb DNA size marker. (**E**) TIDE analysis shows the indel spectrum of the AAVS1 locus after targeting with Cas9 plus PB^dm^ (left panel) and Cas9.PB^dm^ (right panel). (**F**) Sanger sequencing analysis of the targeted region after Cas9.PB^dm^-mediated insertion of promoterless reporter showing two electropherogram traces.

After the successful interplasmid knock-in assay mediated by the TransCRISTI method, we set out to examine its efficiency in site-directed integration in the human genome. For this purpose, we chose the human AAVS1 safe harbor genomic region^15^ (located at the first intron of the PPP1R12C gene on chromosome 19) in HEK293T cells. To verify site-directed integration and to analyze the efficiency, we used a promoterless transposable reporter cassette containing a splice acceptor (SA) site, a sequence encoding T2A self-cleaving peptide, EGFP, and polyA signal (pHDS_600; Table S1). The sgRNA binding site #2 was selected in the first intron of the AAVS1 gene (Fig. 2). Cells were transfected with the plasmid encoding Cas9-PB^dm^, sgRNA #2 (pHX_5004), and the donor plasmid (pHDS_600). By the activity of Cas9-PB^dm^ and the specific sgRNA, the insertion event resulted in the integration of 5′ITR-SA-T2A-EGFP-pA-3′ITR cassette into the first intron of the AAVS1 locus (Fig. 2A). The SA site in the sequence of transcript (produced by the function of the native genomic promoter) resulted in the splicing out of the 5′ITR and attachment of AAVS1 exon 1 to T2A in the transcribed RNA (Fig. 2A). The resulting RNA transcript can now be translated as exon1-T2A-EGFP (Fig. 2B). The percentage of EGFP-expressing cells was calculated over the total number of transfected cells (Fig. 2C). The percentage of EGFP-positive cells in the group transfected with Cas9.PB^dm^, sgRNA #2, and 5′ITR-SA-T2A-EGFP-pA-3′ITR (transposon) was 2.18-fold higher than that in the group of cells transfected with unfused Cas9 and PB^dm^, sgRNA #2, and 5′ITR-SA-T2A-EGFP-pA-3′ITR (Mean of 4.13% vs. 1.89%, respectively; *p* < 0.001) (Fig. 2C). Because of using a promoterless EGFP construct, the calculated percentage of EGFP positive cells revealed the exact number of specific integration events in the cell population. In different control groups, including C1 containing PB^dm^ and transposon, C2 containing Cas9, sgRNA #2, and transposon, and C3 containing Cas9 and transposon, the average number of EGFP-positive cells was 0.26%, 0.4%, and 0.06%, respectively. To verify and examine the expected integration site, the genomic DNA was isolated from transfected cells and was subjected to PCR (using primers F3 and R4, Table S2), gel electrophoresis (Fig. 2D), and Sanger sequencing (Fig. 2F). Thus, the Cas9.PB^dm^ effector was able to perform a sgRNA-guided integration of the promoterless reporter in a specific genomic site. We also compared the efficiency of the Cas9.PB^dm^ effector with that of Cas9 plus PB^dm^ using sgRNA # 2 in the AAVS1 locus with a TIDE assay. As analyzed by TIDE, the Cas9.PB^dm^ effector had a total editing efficiency of 15.8%, while Cas9 plus PB^dm^ generated a total editing efficiency of 8.7% (Fig. 2E).

To compare the efficiency of site-specific insertions in the AAVS1 gene between the two methods of TransCRISTI and CRISPR HITI, 150 clonal cell lines in each group were derived and subjected to genomic PCR. Cells in the TransCRISTI group demonstrated higher efficiency (72%; *n* = 108/150 selected isogenic cells per group) for site-specific insertions than the CRISPR HITI (44%; *n* = 66/150 selected isogenic cells per group) (Table 1). This approach is designed to detect the specific integrations in the AAVS1 gene locus, although there is the possibility of non-specific insertions at other sites of the genome.

**Table 1:** The efficiency of site-specific insertions in the AAVS1 gene using TransCRISTI and CRISPR HITI in selected and derived clonal cell lines.

Method	Total number of isogenic lines	Number of cell lines with specific integration	Percentage of cell lines with specific integration (%)
TransCRISTI	150	108	72
CRISPR HITI	150	66	44

### TransCRISTI is more efficient than CRISPR HITI for specific insertion of a gene into the genome

**Figure 3:**
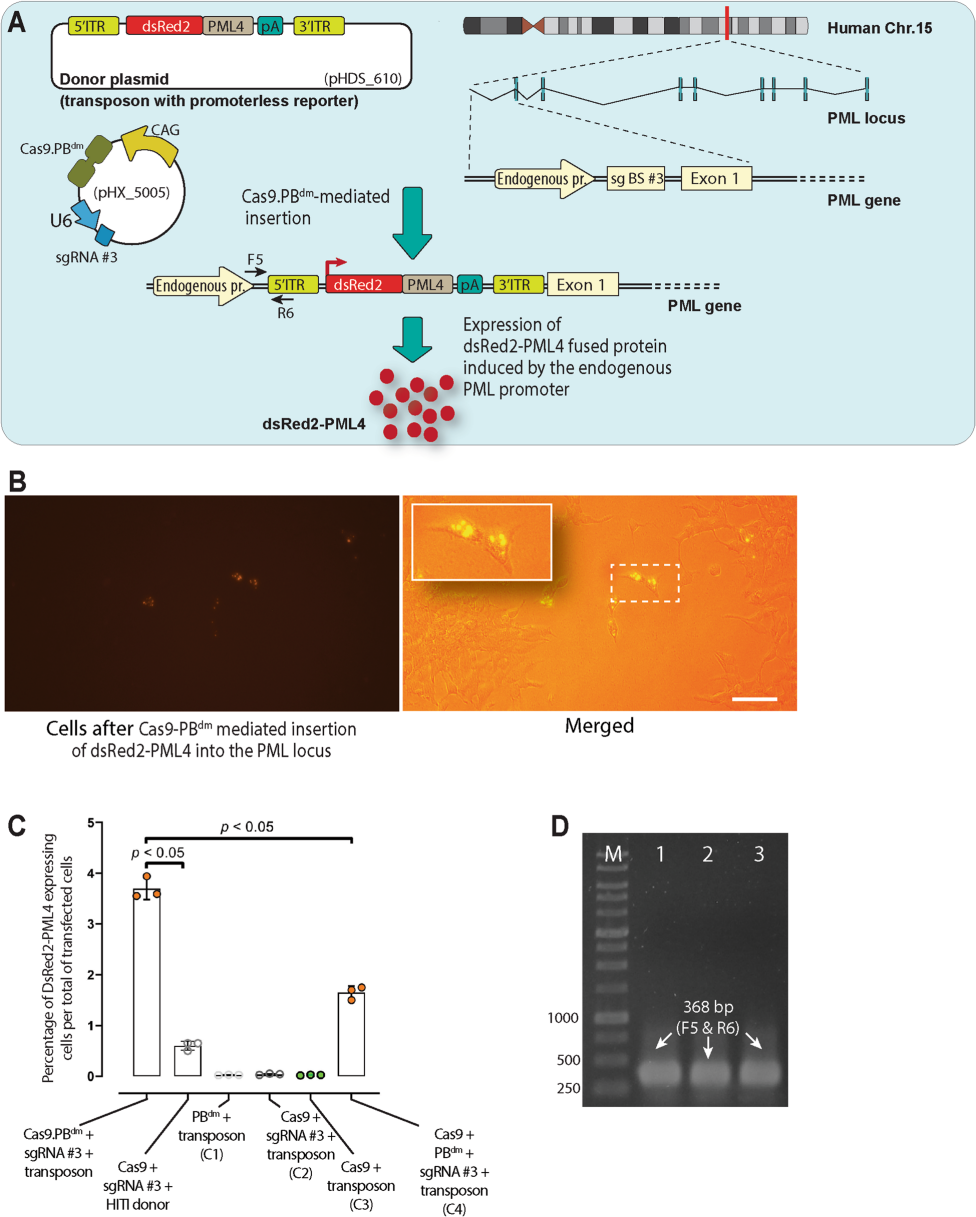
Site-specific TransCRISTI and CRISPR HITI mediated gene knock-in in the PML gene locus. (**A**) Schematic illustration of the TransCRISTI gene knock-in method. The transcription start site (TSS) upstream of the first exon of the PML gene located at chromosome 15 (ACNO. X91752) is targeted by the TransCRISTI method. HEK293T cells were cotransfected with plasmids containing TransCRISTI effector (pHX_5005) and a transposon containing a promoterless DsRed2-PML4 expression cassette (pHDS_610) (Table S1). In the TSS region of the PML gene in chromosome 15, a binding site for sgRNA #3 (sgBS #3) was identified. When the transposon fragment integrates into the sgBS #3, a transcript of DsRed2-PML4 can be produced under the control of the endogenous promoter. In addition, the poly-A signal sequence (pA) in the inserted transposon cassette inhibits transcription of the coding region of the original PML gene in the genome after this transcription termination signal. DsRed2-PML4 protein can be accumulated in PML nuclear bodies as red fluorescent punctations in the nucleus. (**B**) The left panel is the fluorescent image of DsRed2-PML4 expressing cells, 48 h post-transfection. In the right panel which is a merged brightfield and fluorescent image, the inset in the upper left corner shows an enlarged area of the image outlined by a dashed square containing several nuclei with red PML nuclear bodies. The scale bar indicates a length of 100 μm. (**C**) DsRed2-PML4 expressing cells were counted in each experimental group. Group 1 (TransCRISRTI group) cells were transfected with plasmids encoding Cas9.PB^dm^, sgRNA #3, and a plasmid containing the transposon. The plasmid components are shown for the CRISPR HITI group and four control groups (C1, C2, C3, and C4). Results are represented as the percentages of cells with red fluorescent PML nuclear bodies over the total transfected cells. The statistical differences between the TransCRISRTI group and other groups were individually analyzed by the Mann–Whitney U test. *p < 0.05. (**D**) Gel electrophoresis of PCR fragments to confirm the specific knock-in event at the PML locus. 368 bp PCR amplicons in lanes 1, 2, and 3 have been amplified by primers F5 (on the TSS of PML locus) and R6 (on the 5′ITR of the transposon) (Tables S1, S2). Lane M is a 1 kb DNA size marker.

To analyze the applicability and efficiency of the TransCRISTI method for insertion of a gene into a specific region of the genome, we decided to use this technique to insert the coding sequence of PML4 before the transcription start site (TSS) of the PML human locus (ACNO. X91752), and to examine whether this sequence could be transcribed under the activity of the endogenous promoter. This experiment provides a therapeutic model approach for the acute promyelocytic leukemia (APL) disease, in which a translocation results in PML gene knockout. The transposable donor sequence flanked by the PB ITRs was a promoterless DsRed2-PML4-polyA cassette (pHDS_610) (Fig. 3A). Cells were also transfected with the plasmid encoding Cas9.PB^dm^ and sgRNA #3. We identified a sgRNA binding site in the TSS region of the human PML gene in chromosome 15. For CRISPR HITI insertion, a plasmid encoding Cas9 and sgRNA #3, and a donor plasmid with sgRNA binding site #3 were used. Four control groups (C1-C4) were transfected with different combinations of plasmids, PB^dm^ and transposon (C1), Cas9, sgRNA #3, and transposon (C2), Cas9 and transposon (C3), and Cas9, PB^dm^, sgRNA #3 (pHX_5005), and transposon (C4) (Fig. 3). The cells after integration of the gene cassette were identified by the formation of red fluorescent PML nuclear bodies in their nuclei. The appearance of red fluorescent PML nuclear bodies revealed that the inserted promoterless gene cassette has been transcribed under the control of endogenous PML promoter (Fig. 3B). Counting cells with red fluorescent PML nuclear bodies and dividing this number by the total number of transfected cells showed that the percentage of the site-directed integration event in the TransCRISTI group (with a mean of 3.69%) was significantly higher than that of the HITI group (mean: 0.6%) or the C4 group with individual effectors (mean: 1.65%) (Fig. 3C). The mean percentages of DsRed2-PML4 expressing cells in groups C1, C2, and C3 were 0.025%, 0.038%, and 0.023%, respectively (Fig. 3C). The insertion site in the TSS region of the PML gene in chromosome 15 was verified by PCR amplification of a 368 bp PCR product (Fig. 3D) by primers F5 (forward primer designed for the TSS site) and R6 (reverse primer designed for PHDS_610) (Table S2), which was subjected to Sanger sequencing (Fig. S1).

### Functional features of the Cas9.PB^dm^ effector

#### The excisionase activity of the Cas9.PB^dm^ effector

**Figure 4:**
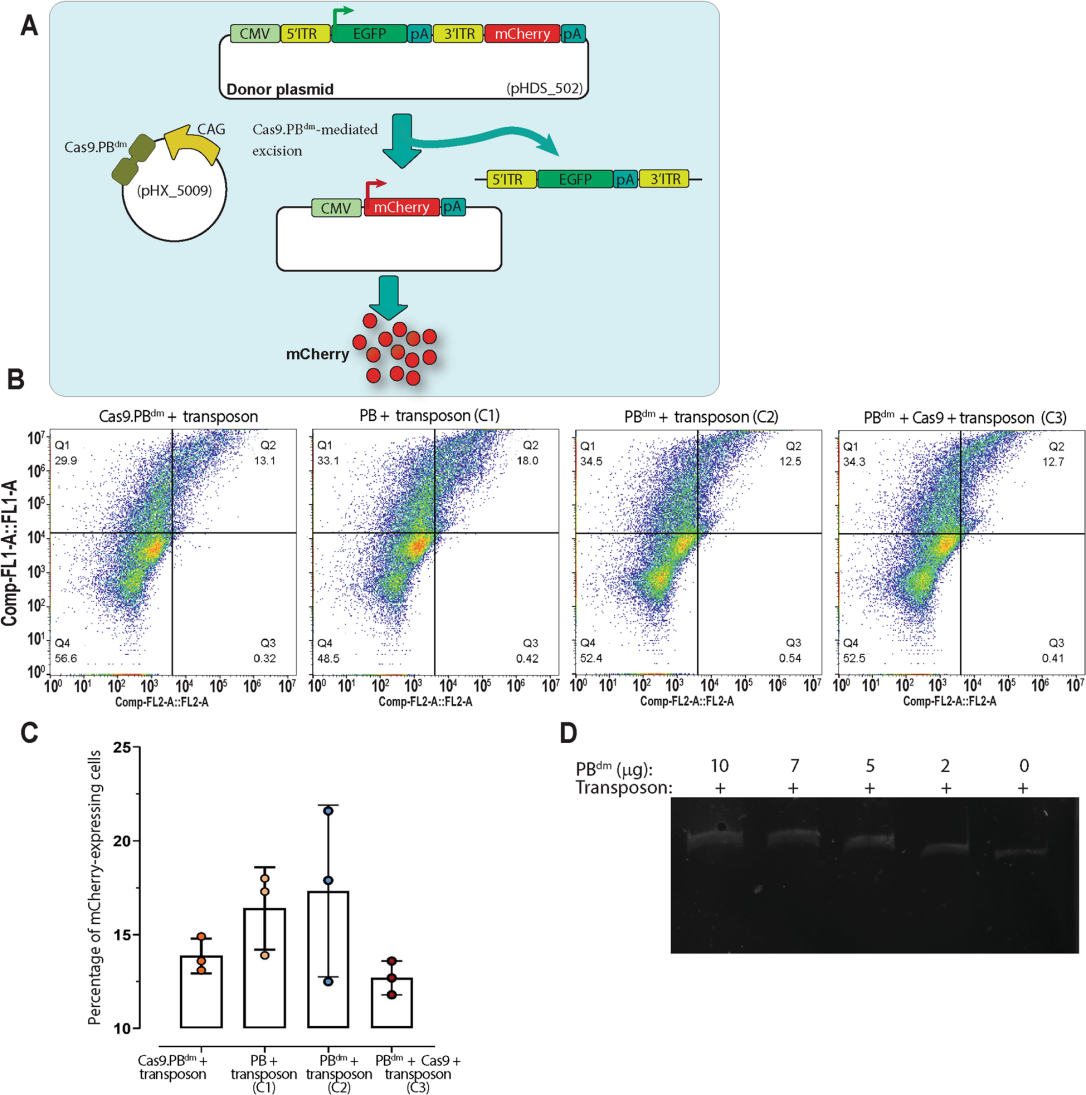
The excisionase activity of PB^dm^. (**A**) A donor plasmid with fragments CMV promoter, 5′ITR, EGFP, poly-A signal (pA), 3′ITR, mCherry, pA was used to evaluate the excisionase activity of PB^dm^ moiety of the Cas9.PB^dm^ molecule. To be able to compare the excisionase activity between PB^dm^ and the wild-type PB, we did not use any sgRNA for targeting. The excision of an EGFP transposable element could result in the activation of expression of mCherry, and a shift from green to red fluorescence. (**B**) Representative flow cytometry analysis images for the main and 3 control groups (C1, C2, C3). Q1: EGFP/mCherry +/− ; Q2: EGFP/mCherry +/+ ; Q3: EGFP/mCherry −/+ ; Q4: EGFP/mCherry −/− . (**C**) The percentage of mCherry-expressing cells acquired by flow cytometry analysis was determined for the main experimental group and 3 control groups. (**D**) EMSA to examine the binding of PB^dm^ to DNA transposon. The purified PB^dm^ enzyme was incubated in increasing concentrations (from 0 to 10 μg for PB^dm^) to 50 ng of a 1200-bp transposon. Then, the retardation in the migration of the PB^dm^ enzyme was imaged.

To evaluate the excisionase activity of PB^dm^ in the Cas9.PB^dm^ effector and to compare that with the excisionase activity of the wild-type PB, an experiment was designed in which the excision of a transposable EGFP cassette could result in the expression of mCherry with red fluorescence (Fig. 4A). The percentages of mCherry expressing cells produced as a result of EGFP transposable element excision were determined 72 h post-transfection by flow cytometry analysis (Fig. 4B). We used three control groups, the C1 control group which was transfected with plasmids encoding the wild-type PB transposase, and the donor/reporter plasmid, the C2 control group transfected with the plasmids encoding the PB^dm^ and the donor/reporter plasmid, and the C3 control group which was transfected with plasmids encoding the PB^dm^ and Cas9 as individual molecules and the donor/reporter plasmid. The percentage of mCherry-expressing cells in the main (TransCRISTI) group and control groups C1, C2, and C3 was determined to be 13.8%, 16.4%, 15.9%, and 12.7%, respectively (Fig. 4C). The statistical analysis showed no significant differences between the main group and three control groups in the number of mCherry-expressing cells as a result of the excisionase activity. In addition, transfection with the only transposon did not result in any mCherry expression (data not shown). These results indicated that the excisionase activity of PB^dm^ moiety of the Cas9.PB^dm^ effector is similar to that in the wild-type PB. Therefore, it can be summed up that the excisionase activity of PB^dm^ has not been significantly affected as the result of mutations in the integration domain of the PB transposase, or by its fusion to the Cas9 protein in the Cas9.PB^dm^ effector.

#### The PB^dm^ moiety in the TransCRISTI effector retains the ability to tether to the transposable element

The ability of the Cas9.PB^dm^ effector in the TransCRISTI method for tethering the transposable element was evaluated by electromobility shift assay (EMSA). The EMSA was performed to check the ability of PB^dm^ for carrying the transposable element after excision. For this purpose, this enzyme was expressed in bacteria and purified. Increasing the concentration of transposase proteins (PB^dm^) caused more shifts in the migration of transposon DNA bands. This assay revealed that PB^dm^ has retained its ability in tethering the transposable element (Fig. 4D). A similar experiment with the wild-type PB resulted in the same findings (data not shown).

#### The Cas9.PB^dm^ effector shows low efficiency for off-target and random insertion of the transposon

**Figure 5:**
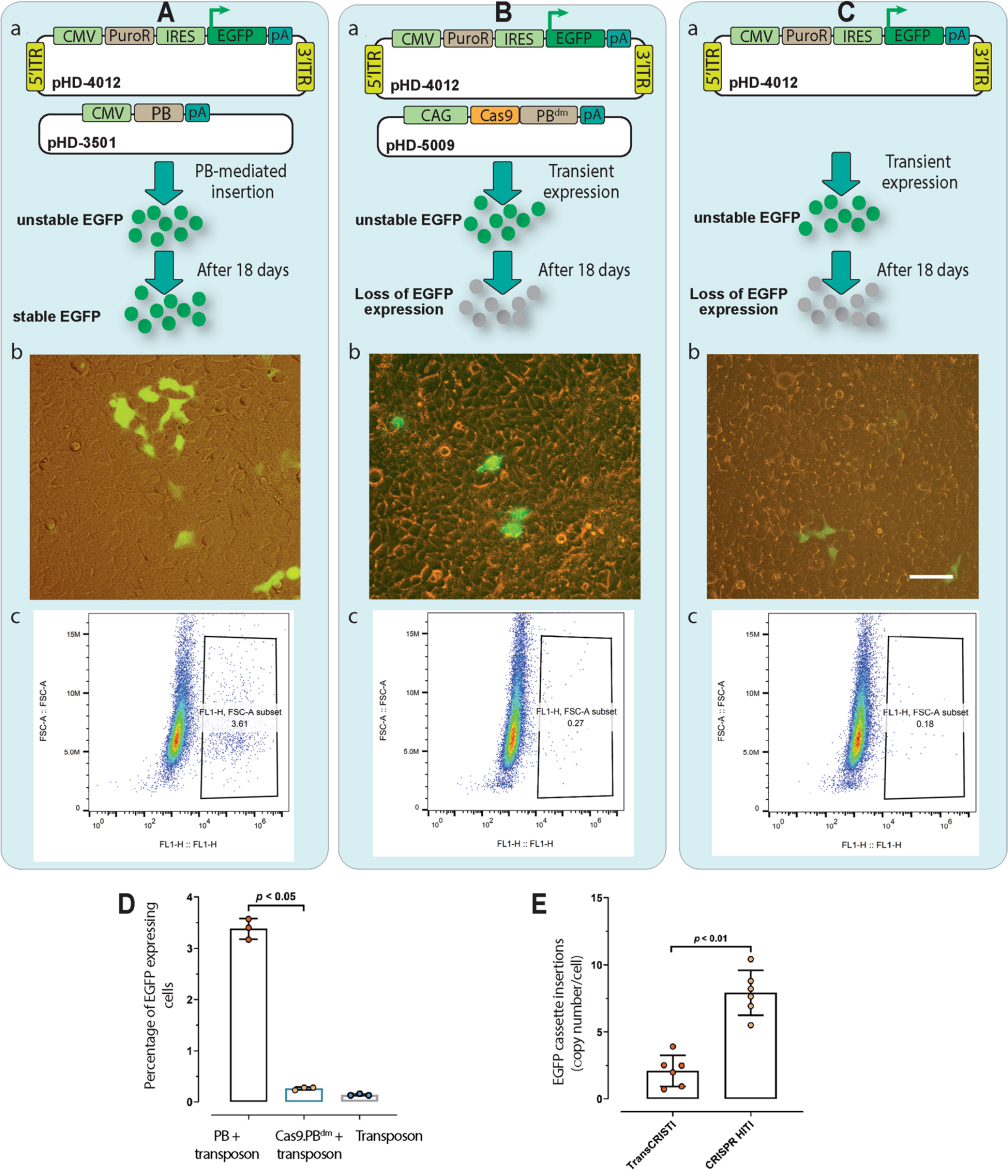
Assay to determine random insertion of the transposon for Cas9.PB^dm^ and wild-type *piggyBac* transposase and evaluation of specific and off-target insertions mediated by the TransCRISTI and CRISPR HITI methods. (**A**) Measurement of random insertion events mediated by wild-type PB. (**B**) Measurement of random insertion events mediated by Cas9.PB^dm^. (**C**) Measurement of random insertion events in cells transfected with only the transposon donor. Panels Aa, Ba, and Ca schematically depict experiments for each group. Panels Ab, Bb, and Cb show merged brightfield and fluorescent images. These representative images show cells that are transfected and cultured for 18 days in each group. The scale bar indicates a length of 100 μm. (**C**) Panels Ac, Bc, and Cc show representative flow cytometry analysis for each group. (**D**) Percentage of stable EGFP-expressing cells determined for each group. (**E**) In isogenic cell lines using qPCR assay the copy number of specific genomic insertion of the EGFP transposable cassette was compared between the TransCRISTI and CRISPR HITI methods. The insertions were calculated relative to the copy number of the EGFP gene in a reference isogenic cell line.

The ability of PB^dm^ moiety of the Cas9.PB^dm^ effector for random insertion of the transposon in the genome was examined and compared with that of the wild-type PB. We reasoned that the determination of the percentage of cells that remain reporter-positive after a period of transient expression would demonstrate the ability of random insertion of the Cas9.PB^dm^ in the absence of any sgRNA. Three groups of cells were transfected (Fig. 5). The positive control group was transfected with the wild-type PB and an EGFP-expressing transposon (Fig. 5A). The main test group was transfected with the Cas9.PB^dm^ and an EGFP-expressing transposon (Fig. 5B). And the negative control group was transfected with only an EGFP-expressing transposon (Fig. 5C). The transfected cells were kept in culture for 18 days to lose the transient expression. Then, they were subjected to flow cytometry analysis. The results showed that the percentage of the EGFP positive cells for the main (TransCRISTI) group was similar to the negative control group, and it was significantly lower than the positive control group (with wild-type PB) (Fig. 5D). These data indicate the very low probability of nonspecific insertion of the transposable element by the Cas9.PB^dm^ effector as a new fusion form of PB^dm^ in comparison with unfused forms of wildtype PB.

To compare the copy number of genomic insertions between the TransCRISTI and CRISPR HITI methods, we performed a qPCR-based copy number assay and determined the number of EGFP transposable cassette insertions for each of six isogenic cell lines (3 lines for each method, approved by Sanger sequencing; Fig. S2). In this assay, the copy number of insertions aside from those in the AAVS1 gene were calculated relative to the copy number of the EGFP gene in an isogenic cell line. In the CRISPR HITI group (n = 3), the average number of transposable cassette insertions was 7.88 copies per cell which were significantly (*p* < 0.05) higher than the average copy number for insertions in the TransCRISTI group (2.055 copies per cell; n = 3) (Fig. 5E).

We also examined and compared possible off-target insertions between the TransCRISTI and CRISPR HITI groups. From each group, 3 isogenic cell lines were subjected to PCR and semi-nested PCR to amplify amplicons extending from any of the five predicted off-target sites into the insert. In the 3 TransCRISTI cell lines, we could not amplify any of the five off-target insertion regions. However, in one CRISPR HITI cell line, we were able to amplify two of the five off-target insertion regions (data not shown). This indicates that off-target insertions are more likely to occur in the CRISPR HITI method than in the TransCRISTI approach in the in-silico predicted sites.

## Discussion

**Figure 6:**
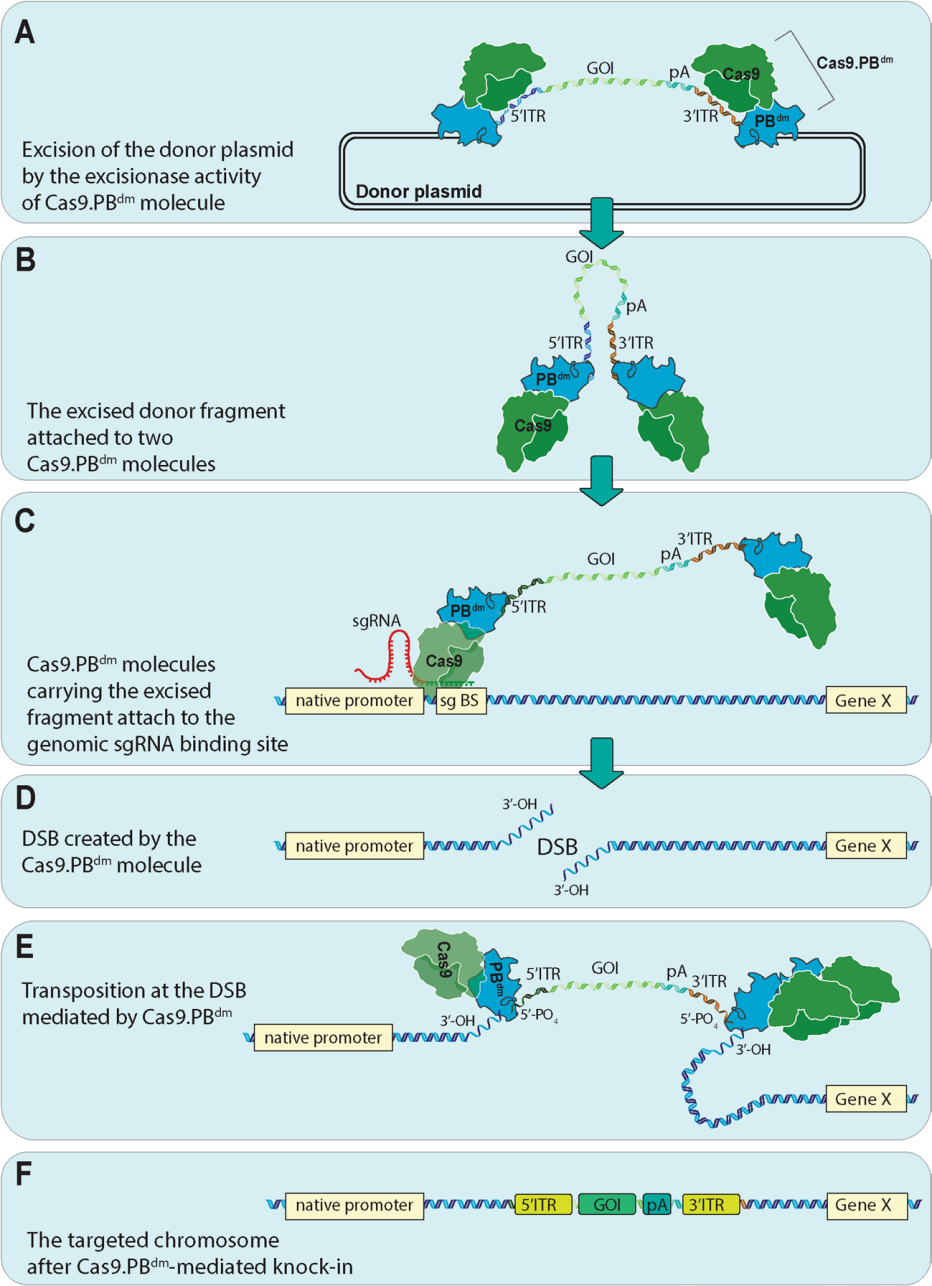
Schematic illustration of TransCRISTI mediated gene knock-in method. The TransCRISTI effector (Cas9.PB^dm^) is composed of a Cas9 endonuclease and an integrase-deficient *piggyBac* transposase which are fused by a peptide linker. The TransCRISTI donor is a PB transposon plasmid carrying a gene of interest (GOI) flanked by the *piggyBac* transposon ITRs. GOI can be a reporter gene (with or without promoter), a selectable marker gene, and a coding sequence for a therapeutic gene. This figure shows one example of the use of the TransCRISTI method for the insertion of a promoterless GOI in a genomic region and its expression under the control of the endogenous (genomic) promoter. (**A**) The excisionase function of TransCRISTI effector molecule. The transposon cassette is excised at the vicinity of the left and right ITRs by the activity of two distinct effector molecules. (**B**) The transposon fragment after excision is attached to a Cas9.PB^dm^ molecule at each end in a U-shaped structure. (**C**) The RNA guiding and nuclease function of the TransCRISTI effector molecule. The Cas9.PB^dm^ molecule is guided by the attachment of complementary sgRNA to a specific sgRNA binding site in the genome. (**D**) A double-strand DNA break (DSB) is created at the guided site by the nuclease activity of Cas9 moiety in Cas9.PB^dm^. (**E**) The available transposon in the vicinity (tethered by the PB^dm^ moiety) will be used for integration in the break site as a repair patch. (**F**) The GOI after this site-specific insertion can be expressed by the activity of the endogenous promoter.

In this study, we have designed and assessed a new site-specific gene knock-in method based on the excision capability of the mutant *piggyBac* transposase and the site-directed genome targeting mediated by the CRISPR/Cas9 system (Fig. 6). Using this method, which we called TransCRISTI, we were able to perform site-directed integration in the AAVS1 safe harbor and PML genomic loci in 4.13% and 3.69% of the transfected cells, respectively (Figs. 2, 3). This method showed higher efficiency and fewer off-target insertions than the CRISPR HITI (One cut donor) in mammalian cells (Table 1, Figs. 3, 5).

Among the transposon systems, PB and *Sleeping Beauty* (SB) are much more widely used as gene transfer tools in human cells^11,16,17^. However, the main impediment for the use of these systems in therapeutic gene integration applications is their semi-random insertions^18^. To obviate the non-specific insertion activity of transposon systems and to perform targeted transposition in predetermined genomic locations, several fusion forms between transposases and site-specific DNA binding domains or programmable site-directed proteins have been developed^19^. The performance of PB transposase fused to ZFP, TALE^10,20^, and GAL4 DBD^21^, and SB transposase fused to ZFP^22^ and GAL4 DBD^23^ have been investigated. In general, these studies have not been able to register site-specific integrations, indicating that as a result of the fusion of the DNA binding domain to transposase, the site-specific insertion capability of transposase does not improve. The relatively small DNA binding domain attached to the large transposase protein could have been the main reason for the failure of the targeted insertions^24^. In some other studies, the performance of the fused form of transposase to the catalytically dead form of Cas9 was examined. The fusion of the wild-type PB and mutant PB (Exc^+^ Int^−^) to dCas9 resulted in low efficiency of site-directed integrations in 0.01% and 0.06% of total cells^10,13^. In a recent study, the fusion of SB to the dCas9 increased the probability of insertion to a 300-bp window nearby the target site which was still far from a precise site-specific insertion event^24^. We have recently shown that by using dCas9 fused to PB and dual sgRNAs, site‐directed integrations could be achieved in 0.32% of transfected cells^14^. However, in all of the above-mentioned studies, the usefulness of these chimeric molecules for gene therapy applications is largely affected by random genomic integrations, inherent to the activity of transposase. Even using several sgRNAs and targeting genomic locations with multiple sgRNA-binding sites has not resulted in a higher number of insertions. In the current study, the introduced chimeric effector molecule (Cas9 fused to the integrase-deficient transposase; Cas9.PB^dm^) was applied on genomic locations with only one sgRNA-binding site and improved the rate of targeted insertions. In a recent study, a fused Cas9 to the DNA binding domain of SB transposase was successful in the unidirectional and specific genomic insertion of long insertions^17^.

In this study, we calculated the efficiency of site-specific integrations using two approaches. In the first approach, we determined the efficiency of site-specific integrations in cells that were selected with an antibiotic. The efficiency of site-specific integration into the AAVS1 gene was calculated to be 72% by this approach (Table 1). Several previously published articles have used a similar approach to determine the efficiency of site-specific insertions^10,13,20,25^. In the second approach similar to our previous report^14^, we used the promoter/reporter complementation assay to register and recover cells harboring specific integrations. In this strategy, the promoterless reporter fragments can only be activated (by the endogenous promoters) when they are placed precisely in the integration site. This complementation of the endogenous promoter and the integrated reporter to register and detect the positive targeting insertions^14^, is a stringent method to determine the accurate targeting events that are solely dependent on site-directed integration. Using this approach, from the initially transfected population, we determined site-directed integration in the human AAVS1 safe harbor genomic region in 4.13%, and in the PML locus in 3.69% of cells. To register cells with the desired integrations in this method there is no need for the initial positive selection of cells with an antibiotic drug that results in the loss of the original untransfected cells, which in turn could lead to incorrect calculation of the percentage of positive cells out of the original population. Other studies have used donor fragments that contain reporter genes that can be expressed independently (out of the insertion sites) and thus, can lead to false-positive results^10,13,20,21,26^.

Using the promoter/reporter complementation assay, the integration ability of the Cas9.PB^dm^ was evaluated in inter-plasmid and genome site-specific insertions in the AAVS1 and PML loci (Figs. 1, 2, 3). The percentage of DsRed2-PML4 expressing cells in the TransCRISTI group was significantly higher than that in the group subjected to CRISPR HITI with the same donors (Fig. 3C). Interestingly, the usage of two separated moieties of Cas9 and PB^dm^ resulted in higher efficiency of gene knock-in than that of the CRISPR HITI method (Fig. 3C). The efficient excision and linearization of the donor fragment by the excisionase activity of PB^dm^ compared to the nuclease activity mediated by Cas9 in the HITI method may partly explain these results. In TransCRISTI, the donor construct is designed with asymmetric ends, similar to that in the PB transposition elements^27^, and the transposable donor does not need any sgRNA-binding sites for in vivo linearization contrary to the CRISPR HITI method^8,28^. PB transposase acts in an asymmetric dimer in which a highly conserved Cysteine-Rich Domain (CRD) at the C-terminal of each PB dimer, contacts only one asymmetric transposon end (ITR)^12^. Furthermore, unlike the donor fragments in HR-dependent methods, the TransCRISTI method does not need any homology arms on its donor element^28^. Thus, the TransCRISTI donor is a transposon-based element that can be designed site-independently and can be used as a general donor construct. This transposon-based donor also can carry large (> 200 kb) DNA cargos^29^. Another explanation for the better efficiency of TransCRSITI than HITI can be the capacity of PB to connect (tether) and carry the excised donor fragment and to facilitate the integration of foreign gene fragment in the target area^30,31^. We used EMSA assay to assess the protein–DNA interactions of PB^dm^ and the tethering feature of the Cas9.PB^dm^ (Fig. 4D). We found that the PB^dm^ has retained its ability to tether the transposable element (Fig. 4D). Thus, it seems that PB^dm^ can make a complex with the excised transposon element and carry it to the target region.

This higher efficiency of performance in the Cas9.PB^dm^ fused molecule could be related to each or a combination of molecular abilities of excision, tethering, and integration in the Cas9.PB^dm^ effector. The chimeric effector molecule developed in this study contains a mutant PB, fused to Cas9. The mutant PB with two mutations of R372A and K375A (PB^dm^) has kept its excision capacity (Fig. 4), while it has lost the connections to the target site and subsequent bending at the TTAA tetra-nucleotide targeting sites, leading to the lack of integration ability (Fig. 6)^12,27^. The excisionase activity of the PB^dm^ fused to Cas9 was slightly (2.1%) and non-significantly lower than that in the unfused PB^dm^. This was assessed by the efficiency of 5′ITR-EGFP-PA-3′ITR excision from a plasmid, leading to the rejoining of CMV and mCherry in the plasmid, and the expression of the red reporter (Fig. 4A). Also, the excisionase activity of the fused mutant PB, assessed in comparison with that in the wild-type and unfused PB, showed that this activity was independent of the function of sgRNA (Fig. 4B,C).

The Cas9. PB^dm^ resulted in significantly lower off-target insertions than CRISPR HITI (Fig. 5E). A comparison between the copy number of inserted fragments at the TransCRISTI and CRISPR HITI exhibited that the inserted copies in the HITI strategy were around 3.83-fold higher than that in the TransCRISTI strategy (Fig. 5E). The higher copy number of fragments can be the result of higher off-target events in the HITI strategy. One possible explanation for the lower off-target and random insertions mediated by the Cas9.PB^dm^ molecule could be that the fusion of Cas9 to the transposase has increased the molecular weight of the fused molecule to around 227 kD, possibly reducing the dwell-time for off-target regions^32^. Perhaps the increased dwell-time of the Cas9.PB^dm^ effector can also explain the 1.81-fold higher editing efficacy (in terms of indels) of this molecule compared with the unfused Cas9 (Fig. 2E). The second explanation for the lower off-target and lower random insertions mediated by the Cas9.PB^dm^ molecule can be a diminished integration ability of the mutant version of the PB transposase that resulted in significantly lower random integrations (and similar to that in the no-effector control group) than the wild-type PB (Fig. 5D).

We used the TransCRISTI gene knock-in method to insert a PML coding sequence in front of the PML native promoter to present a therapeutic model approach for the acute promyelocytic leukemia (APL) disease, in which a translocation results in PML gene knockout. Around 95% of the APL patients show a chromosomal translocation (15;17) leading to the formation of PML-RARa chimeric protein which will cause leukemogenesis^33^. The TransCRISTI mediated targeted insertion at the PML locus resulted in the expression of the inserted PML coding sequence under the control of the PML endogenous regulatory elements in 3.69% of total transfected cells (Fig. 3). Upregulation of PML leads to reduced cell proliferation and migration, apoptosis, and oncogenesis, so it is important to use an endogenous promoter and native regulatory elements for the expression of the therapeutic genes inserted in the genome^34^.

The gene knock-in method developed in this study, named TransCRISTI, was applied and evaluated for site-specific integration into a human safe harbor region and the PML locus. The site-directed insertion efficiency of TransCRISTI was compared with the most eligible mammalian CRISPR-based gene knock-in method, CRISPR HITI. The TransCRISTI method demonstrated higher efficiency of site-specific integration, and lower random and off-target integration than the CRISPR HITI method. Furthermore, the TransCRISTI method provides the possibility of using universal donor constructs and cargos with large sizes. The universal structure of the donor construct in this method makes the TransCRISTI a good candidate for multiple gene insertions in the genome using multiple sgRNAs. TransCRISTI can be considered as a candidate method for future gene therapy applications.

## Materials and methods

### Construction of plasmids and engineering of mutated PB transposase fused to Cas9

Different plasmids (Table S1) were constructed using PCR, gene synthesis, and restriction enzyme-based subcloning methods. The constructs developed in this study are freely available without licensing restrictions to other researchers and nonprofit academic users. The sequences of all constructs were confirmed by Sanger sequencing (Macrogen Inc., South Korea). The constructs expressing effector enzymes/sgRNAs (Table S1, section a) including constructs for expressing the wild-type *piggyBac* (WT PB) transposase (pHX_3501), the double mutant *piggyBac* (PB^dm^) transposase (pHX_5001), the fused Cas9 to PB^dm^ (Cas9.PB^dm^) (pHX_5002, _5004, _5005, _5009) effector, and Cas9 endonuclease (pHX_4591, _4592, and _4593) were generated. To make the TransCRISTI effector construct (encoding Cas9.PB^dm^; pHX_5002, _5004, _5005, _5009), a 156-nucleotide linker sequence including two SV40 nuclear localization sequences (NLS) between the spCas9 (Streptococcus pyogenes type2 CRISPR/Cas endonuclease) and PB^dm^ coding sequences were used (Table S1). PB^dm^ (Exc^+^/Int^−^) transposase was generated by introducing R372A and K375A mutations in the catalytic core region of the codon-optimized PB as previously described^12^. Site-directed mutagenesis by overlap extension with PCR was used to generate the mutant PB (PB with R372A/K375A). Briefly, to generate PB^dm^, three separate PCR reactions using Phusion polymerase in HF buffer (Thermo Fisher Scientific, Waltham, MA, USA) were performed. Two primary PCR reactions with two pairs of primers, mPB-F1 with mPB-R1 and mPB-F2 with mPB-R2, produced two mutant fragments (1304 bp and 706 bp, respectively) with overlapping ends. Then, a second PCR reaction with one pair of primers (mPB-F1, mPB-R2) was used to join the two fragments (linker + PB^dm^; equal to 1978 bps).

The MIT CRISPR design tool (http://crispr.mit.edu/) was used to design sgRNAs. The corresponding DNA motifs were synthesized and cloned in appropriate plasmids as detailed below. The single guide RNA (sgRNA) sequences targeting a plasmid locus (sgRNA #1) and different human gene loci (sgRNA #2 and sgRNA #3) were cloned in different effector plasmids (Table S1) to be expressed under the control of human U6 promoter (hU6). For cloning the fragments encoding sgRNAs, two single-strand DNA guide sequences were mixed in equimolar concentrations (100 pmol/μl) and annealed in a thermocycler using the program 95 °C for 2 min followed by gradual cooling to 25 °C in 45 min, in the annealing buffer (10 mM Tris, 1 mM EDTA, 50 mM NaCl, pH 7.5–8.0). Then, the annealed fragments for sgRNAs #1, #2, and #3 with overhangs were ligated to BbsI-linearized constructs (Table S1). The annealed fragment providing the sgRNA binding site (+ PAM) for sgRNA #3 was ligated into the PvuI-linearized CRISPR HITI plasmid pHDS_612.

Plasmids carrying the transposable elements contained the left (5′ITR) and right (3′ITR) PB terminal repeats flanking different reporter cassettes or promoters (Table S1, section b) (Table S1, section b). The expression plasmids for the inducible bacterial expression of transposase molecules were cloned in-frame with pH-dependent NpuC (C-fragment of the split intein derived from *Nostoc punctiforme*)^35^ (Table S1).

### Cell culture and transfection

Human Embryonic Kidney (HEK) 293 T cell line (ATCC_CRL-11268) was purchased from the National Cell Bank of Iran (Pasteur Institute, Iran). HEK293T cells were cultured in DMEM (Gibco, USA) supplemented with 10% heat-inactivated fetal bovine serum (Gibco, USA) at 37 °C with 5% CO_2_ atmosphere. One day before transfection, cells were seeded at the density of 5 × 10^4^ cells per well in 24-well plates. The following day the cells were transfected with appropriate plasmids as detailed below. For each well of 24-well plate, 2 μg of plasmid DNA was diluted in 50 μl of HEPES buffer (20 mM HEPES, pH 7.4) and incubated for 5 min at room temperature. In the transfection of groups with a lower number of plasmids, we used the pBluescript II SK (+) plasmid to equalize the amount of plasmid DNA for each group. In addition, we applied an equal copy number of the main plasmids among different transfection groups. In a separate reaction, 50 μl of HEPES buffer was added to 2.5 μl of 1 mg/ml of 25 KDa branched Polyethylenimine (bPEI25; Sigma-Aldrich, USA) and mixed briefly. The DNA preparation was added to the diluted polymer solution and vortexed mixed and incubated at room temperature for 25 min to allow the formation of the polymer-DNA complex (polyplex). The resulting polyplex was diluted in 400 μl of prewarmed Opti-MEM (transfection medium) (Gibco, USA), incubated for 5 min, and then used to replace the existing medium on the cells. Cells were incubated at 37 °C for 6 h before changing the transfection medium to a normal culture medium.

### Flow cytometry analysis

The transfected cells were washed with cold phosphate-buffered saline (PBS) and then detached using 0.25% trypsin/EDTA solution (Biowest, France). Detached cells were washed three times in PBS. Then, 200,000 cells were subjected to flow cytometry analysis (BD FACSCalibur, USA). The proportion of EGFP-expressing, or mCherry-expressing cells was then determined. The data were analyzed with FlowJo version 7.0 software.

### Interplasmid knock-in assay

To perform an interplasmid knock-in assay, 5 × 10^4^ HEK293T cells were transfected with 2 μg of plasmids encoding the Cas9.PB^dm^ effector and sgRNA #1 (pHX_5002; Table S1), a donor plasmid containing 5′ITR-CMV promoter-3′ITR (pHD_40191), and an acceptor plasmid containing a sgRNA binding site for sgRNA #1 and a promoterless reporter DsRed2-PML4-SV40 PolyA (pHDF_8001). In the first control group, Cas9.PB^dm^ was replaced with only Cas9. In the second control group, Cas9.PB^dm^ was replaced with only PB^dm^. In the third control group, Cas9.PB^dm^ was replaced with Cas9 in the absence of sgRNA #1 (pHX_459). And, in the fourth control group, Cas9.PB^dm^ was replaced with Cas9 and PB^dm^ as separate molecules. 72 h after transfection, cells that expressed the red PML nuclear bodies were counted as DsRed2-PML4 positive cells by fluorescence microscopy. In Fig. 1C, each dot represents the percentage of DsRed2-PML4 positive cells which has been calculated by dividing the number of DsRed2-PML4 positive cells by the total number of transfected cells in one well of a 24-well plate. The total number of transfected cells (with an equal mass of plasmids containing a fluorescent construct) was determined 72 h after transfection by flow cytometry analysis. These experiments were performed in triplicates. To identify the exact location of insertion in the acceptor plasmid, 72 h after transfection cells were subjected to plasmid extraction. The correct location of transposon insertion into the sgRNA binding site, upstream of DsRed2-PML4 in the acceptor plasmid was validated using PCR amplification of the insertion site on plasmid DNA by primers F1 and R2 (Table S2). PCR program included 4 min at 95 °C (1 ×), followed by 30 s at 95 °C, 30 s at 60 °C, and 30 s at 72 °C (35 ×). PCR amplicon was validated by restriction enzyme digestion (AgeI; Thermo Fisher Scientific, Waltham, MA, USA) and Sanger sequencing.

### Assays for genomic integration in the AAVS1 safe harbor region

To evaluate the TransCRISTI-mediated genomic integration in the AAVS1 gene, 5 × 10^4^ HEK293T cells were transfected with 2 μg of plasmids encoding the Cas9.PB^dm^ effector and sgRNA #2 (pHX_5004; Table S1), and a donor plasmid providing the transposon cassette containing in order 5′ITR, the splice acceptor site (SA), a sequence encoding the T2A self-cleaving peptide, the EGFP gene, the poly-A signal, and 3′ITR (pHDS_600). The C1 control group was transfected with the plasmid encoding PB^dm^ (pHX_5001) and a plasmid providing the transposon cassette (pHDS_600). The C2 control group was transfected with the plasmid encoding Cas9 and sgRNA #2 (pHX_4593), and the plasmid providing the transposon cassette (pHDS_600). The C3 control group was transfected with similar plasmids as the C2 group without sgRNA. And, the C4 control group of cells was transfected with plasmids encoding the unfused Cas9 and PB^dm^ effector molecules, sgRNA #2 (pHX_4593, pHX_5001), and the plasmid providing the transposon cassette (pHDS_600). 48 h post-transfection, cells were observed under a fluorescence microscope (Nikon, ECLIPSE Ts2R-FL, Nikon Co., Tokyo, Japan). 10 microscopic views were randomly selected and imaged in each group after 48 h. All cells within one well were counted and since there was an approximately equal number of cells in different wells, there was no need to normalize the number of counted cells in each group. In Fig. 2C, each dot shows the percentage of EGFP-positive cells which has been calculated by dividing the number of EGFP cells by the total number of transfected cells. The total number of transfected cells (with an equal mass of plasmids containing a fluorescent construct) was determined 72 h after transfection by flow cytometry analysis. In addition, the number of EGFP positive cells was normalized by subtracting the number of green cells from the number of positive cells in the control group. Each calculation was repeated in three separate transfections. The total number of transfected cells was estimated by counting the EGFP-positive cells that were transfected in a different well with a similar construct, albeit with a promoter. To identify the exact location of insertion in the genome, 72 h after transfection cells were subjected to the extraction of genomic DNA. The insertion into the sgRNA binding site in the intron of the AAVS1 gene was identified using PCR amplification of the genomic DNA by primers F3 and R4 (Table S2). PCR program included 4 min at 95 °C (1 ×), followed by 30 s at 95 °C, 30 s at 60 °C, and 30 s at 72 °C (35 ×).

### TIDE analysis

HEK293T cells were seeded at the density of 5 × 10^4^ per well of a 24-well plate. The next day, cells were transfected in two test groups: in the first test group the plasmid pHX_5004 (encoding sgRNA #2 for targeting the human AAVS1 gene locus and Cas9.PB^dm^), and in the second test group plasmids pHX_5001 (encoding mutant *piggyBac*) and pHX_4593 (encoding sgRNA #2 for targeting the human AAVS1 gene locus and Cas9) were transfected. As a control group, we used untransfected HEK293T cells. After 72 h, all the cells in test and control groups were harvested and genomic DNA was isolated using the Animal DNA Isolation Kit (DENAzist Asia Co., Mashhad, Iran). Then, the AAVS1 locus was amplified using primers F3 and R17, (Table S2). PCR program included 4 min at 95 °C (1 ×), followed by 30 s at 94 °C, 30 s at 60 °C, and 20 s at 72 °C (35 ×). PCR products in all sample and control groups were column-purified and subjected to Sanger sequencing (Macrogen Inc., Seoul, South Korea) by using the primer R17. Then, all the sequences were analyzed using the TIDE online tool (https://tide.nki.nl/)^36^.

### Assays for genomic integration in the PML gene locus

To evaluate genomic integration in the TSS site of the PML gene, 5 × 10^4^ HEK293T cells were transfected with plasmids encoding the Cas9.PB^dm^ effector and sgRNA #3 (pHX_5005, 1000 ng; Table S1), and a donor plasmid providing the transposon cassette containing in order 5′ITR, the fused DsRed2-PML4 coding region, the poly-A signal, and 3′ITR (pHDS_610, 1000 ng). The CRISPR HITI group was transfected with the plasmid encoding Cas9 and sgRNA #3 (pHX_4591) and a plasmid providing the one cut HITI donor cassette containing a sgRNA binding site for sgRNA #3 (pHDS_612). The C1 control group was transfected with the plasmid encoding PB^dm^ (pHX_5001) and a plasmid providing the transposon cassette (pHDS_610). The C2 control group was transfected with the plasmid encoding Cas9 and sgRNA #3 (pHX_4591), and the plasmid providing the transposon cassette (pHDS_610). The C3 control group was transfected with similar plasmids as the C2 group without sgRNA. And, the C4 control group of cells was transfected with plasmids encoding the unfused Cas9 and PB^dm^ effector molecules, sgRNA #3 (pHX_4591, pHX_5001), and the plasmid providing the transposon cassette (pHDS_610). 48 h post-transfection, cells were observed under a fluorescence microscope (Nikon, ECLIPSE Ts2R-FL, Nikon Co., Tokyo, Japan). In Fig. 3C, each dot represents the percentage of cells with red fluorescent PML nuclear bodies which were calculated by dividing the number of these cells by the total number of transfected cells. The total number of transfected cells (with an equal mass of plasmids containing a fluorescent construct) was determined 72 h after transfection by flow cytometry analysis. All cells with red fluorescent PML nuclear bodies in a well were counted, and these experiments were performed in triplicates. The total number of transfected cells was estimated by counting the DsRed2-positive cells that were transfected in a different well with a similar construct, albeit with a promoter. To identify the exact location of insertion in the genome, 72 h after transfection cells were subjected to the extraction of genomic DNA. These experiments were performed in triplicates. The correct location of insertion into the sgRNA binding site in the TSS region of PML was identified using PCR amplification of the genomic DNA by primers F5 and R6 (Table S2). PCR program included 4 min at 95 °C (1 ×), followed by 30 s at 95 °C, 30 s at 60 °C, and 30 s at 72 °C (35 ×). PCR amplicon was validated by Sanger sequencing.

### PCR recovery of the targeted regions

For the evaluation of all genomic insertions, genomic DNA was isolated using the Animal DNA Isolation Kit (DENAzist Asia Co., Mashhad, Iran). Plasmid isolations from bacteria and in the interplasmid knock-in assay from HEK293T cells were performed using the Plasmid Isolation Kit (DENAzist Asia Co., Mashhad, Iran). An approximately 600 bp region immediately upstream or downstream of the insertion regions was amplified in the genomic and plasmid DNAs using the Taq DNA Polymerase 2 × Master Mix RED (Amplicon, Denmark). PCR amplicon from the 5′-flanking region of the integrated site in the AAVS1 and PML genes was validated by Sanger sequencing. We did not amplify the 3′flanking region for these regions. All primers were designed using oligo7 software (version 7.57) (Table S2). The amplified products were gel extracted by the Gel Extraction Kit (DENAzist Asia Co., Mashhad, Iran), and were subjected to Sanger sequencing (Macrogen Inc., Seoul, South Korea).

### The excisionase activity assay

For the excisionase activity assay, a donor/reporter plasmid was constructed that contained an EGFP transposon in between a CMV promoter and a mCherry coding sequence (pHDS_502). In the main (TransCRISTI) group, 5 × 10^4^ HEK293T cells were transfected with plasmids encoding the Cas9.PB^dm^ effector without sgRNA (pHX_5009, 1000 ng; Table S1), and the donor/reporter plasmid (pHDS_502, 1000 ng). Three control groups were also transfected. The C1 control group was transfected with plasmids encoding the wild-type PB transposase (pHX_3501), and the donor/reporter plasmid pHDS_502. The C2 control group was transfected with the plasmids encoding the PB^dm^ (pHX_5001), and the donor/reporter plasmid pHDS_502. The C3 control group was transfected with plasmids encoding the PB^dm^ (pHX_5001) and Cas9 (pHX_459) as individual molecules, and the donor/reporter plasmid pHDS_502. 72 h post-transfection, we analyzed EGFP and mCherry expression using flow cytometry analysis and fluorescence microscopy. Removal of the EGFP by the excisionase activity of PB^dm^ and wild-type PB could result in the expression of mCherry. This experiment was performed in triplicates.

### Electrophoretic mobility shift assay (EMSA) for the evaluation of transposon tethering ability of the mutant *piggyBac* transposase

To evaluate the transposon tethering ability of the PB^dm^, this enzyme was expressed and purified from bacterial extracts. Then, it was incubated with the transposon donor plasmid and subjected to electrophoretic mobility shift assay (EMSA). The coding sequence of PB^dm^ was cloned in a bacterial expression plasmid, C-terminal to the pH-dependent NpuC (C-fragment of the split intein derived from *Nostoc punctiforme*)^35^. The BL21(DE3) bacterial cells containing this plasmid were cultured and induced with 1 mM IPTG (Isopropyl β- d-1-thiogalactopyranoside). Another plasmid containing elastin-like peptide (ELP) fused to the pH-dependent NpuN (N-fragment of the split intein derived from *Nostoc punctiforme*) was transformed into BL21(DE3) cells and after culture to the required optical density were induced for the expression of ELP-NpuN. Bacterial cell lysates after sonication and clarification were mixed. By adding ammonium sulfate to a final concentration of 0.4 M, the protein products (NpuC-PB^dm^ attached to ELP-NpuN) were precipitated. The precipitates were dissolved in cleaving buffer and were incubated for 5 h at 37 °C to induce the cleavage of PB^dm^. A final addition of ammonium sulfate to a final concentration of 0.4 M was used to precipitate the remaining proteins. The supernatant contained PB^dm^.

Then, the purified protein with different concentrations (2, 5, 7, and 10 ug/ul) was incubated with a 1200 bp PB transposon (50 ng) in the binding buffer at 30 °C. The DNA transposon was a linear construct acquired from PCR amplification of 5′ITR-CMV enhancer-3′ITR *piggyBac* transposon from the plasmid (PHD_4015) by primers F15 and R16 (Table S2). The composition of binding buffer was 25 mM HEPES (pH 8.0), 3 mM Tris (pH 8.0), 75 mM NaCl, 2 mM DTT, 10 mM MgCl_2_, 0.01% BSA, and 3.75% glycerol. After incubation in the binding buffer for 40 min, samples were added to the loading buffer (6 ×) consisting of 1 mM EDTA, 10 mM Tris, 50% (v/v) glycerol, 0.001% (w/v) bromophenol blue and 0.001% (w/v) Xylene cyanol. Then, samples were loaded on a 0.5% native polyacrylamide gel. Electrophoresis was performed at 100 V at 4 °C in 1 × TAE gel for 40 min. The gel was visualized by post-staining using ethidium bromide (0.5 µg/ml) in the TAE buffer.

### Assay to determine random insertion of the transposon mediated by Cas9.PB^dm^

To evaluate the random insertion activity of PB^dm^ moiety of the Cas9.PB^dm^ effector in comparison with the activity of wild-type PB, we set out to determine the percentage of cells that remain reporter-positive after a period of transient expression. For this purpose, the positive control group was transfected with the plasmid encoding the wild-type PB (pHX_3501) and a donor plasmid containing 5′ITR-CMV-PuroR-IRES-EGFP-pA-3′ITR transposon (pHD_4012). The main test group was transfected with the plasmid encoding the Cas9.PB^dm^ effector without sgRNA (pHX_5009) and the donor transposon plasmid (pHD_4012). One negative control group was also transfected with only the donor plasmid pHD_4012. After the transfection of HEK293T cells, cells were cultured and maintained for 18 days to lose their transient EGFP expression. Eighteen days post-transfection, we quantified EGFP expression using flow cytometry analysis. Transfections for all groups and the readings were performed in triplicates.

### Measurement of site-specific insertions in clonal cell lines

For the TransCRISTI group, HEK293T cells were transfected with plasmids encoding the Cas9.PB^dm^ effector and sgRNA #2 (pHX_5004), and a donor plasmid containing 5′ITR-CMV-PuroR-IRES-EGFP-pA-3′ITR (pHD_4012). For the HITI group, HEK293T cells were transfected with plasmids encoding Cas9 and sgRNA #2 (pHX_4593), and a donor plasmid containing 5′ITR-CMV-PuroR-IRES-EGFP-pA-3′ITR (pHD_4012). After 48 h, the transfected cells were selected with puromycin sulfate (0.7 µg/ml; Sigma-Aldrich, USA). In each group, 150 EGFP-expressing cells were selected for isogenic expansion. After expansion, the isolated positive cells in each group were subjected to PCR amplification of the genomic DNA by primers F3 and R4 (Table S2) to determine specific integration in the AAVS1 gene. PCR program included 4 min at 95 °C (1 ×), followed by 30 s at 95 °C, 30 s at 60 °C, and 30 s at 72 °C (35 ×). This PCR assay detects integration in the AAVS1 gene. However, using this technique, we can not rule out or detect any possible non-specific insertions.

### Comparison of insertion copy numbers between the TransCRISTI and CRISPR HITI knock-in methods

Three isogenic cell lines from each group (TransCRISTI and CRISPR HITI) were used to evaluate the copy number of knock-in events. A clonal cell line with the EGFP gene insertion was used as a reference cell line. As a negative control, we used untransfected HEK293T cells. Genomic DNA from six isogenic cell lines, as well as the reference and control cell lines was isolated using the Animal DNA Isolation Kit (DENAzist Asia Co., Mashhad, Iran). We used qPCR assay to compare the copy number of EGFP fragments in the genome of each isogenic cell line (using primers F17 and R18, Table S2). Reactions were performed in a Rotor-Gene Q real-time PCR cycler (Qiagen, USA). PCR program included 10 min at 95 °C (1 ×), followed by 30 s at 94 °C, 20 s at 62 °C, and 10 s at 72 °C (38 ×), followed by a melt curve analysis from 45 °C to 95 °C at 1 °C/10 s. Real-time readings for each group were performed in triplicates. A standard curve for the EGFP gene was generated from the control isogenic EGFP cell line and was used as a reference to compare the EGFP gene copy number in different TransCRISTI or CRISPR HITI isogenic cell lines. The cycle threshold (Ct) numbers for each reaction were used to calculate the copy number of EGFP insertions from the standard curve. The amount of input gDNA in each reaction of qPCR was determined by an RNase P standard curve. RNase P is considered a standard gene, known to be in two copies in the human genome. The qPCR reactions to calculate the copy number of RNase P gene in the genome of cells (using primers F19 and R20, Table S2), were performed by the program 10 min at 95 °C (1 ×), followed by 30 s at 94 °C, 30 s at 65 °C, and 30 s at 72 °C (38 ×), followed by a melt curve analysis from 50 to 95 °C at 1 °C/10 s.

### Assay for off-target insertions

To examine the possibility of off-target insertions, the cells that have been transfected with TransCRISTI (plasmids pHX_5004, pHD_4012) or CRISPR HITI (plasmids pHX-4593 pHD_4012) were selected with puromycin sulfate (0.7 μg/ml; Sigma-Aldrich, USA) for 18 days after transfection. After antibiotic selection, 100 single EGFP positive cells from both groups were isolated and cultured to expansion. Forward primer (primer F3) located at the AAVS1 human locus and reverse primer (primer R4) at the 5′ITR of the transposon (Table S2), were used for PCR amplification and confirmation of genomic insertions in the AAVS1 locus. Three confirmed isogenic lines in each group were subjected to genomic DNA isolation and evaluated for off-target insertions. The search for the possible off-target binding sites of sgRNAs used in the TransCRISTI and CRISPR HITI was performed using a web server for selecting rational CRISPR/Cas9 targets (CRISPRdirect, https://crispr.dbcls.jp). Five possible off-target sites in the human genome were selected. For insertion in these sites, PCR primers were designed (primers for off-target genomic sites F8-12) (Table S2). Then, for each clone, the first round of PCR (using primer R13) and semi-nested PCR reactions (using primer R14) were performed to amplify any of the 5 possible target sites. The specific location of predicted insertion sites and amplification results are summarized in Table S3.

### Statistical analysis

Data were summarized and analyzed using GraphPad Prism 6.0 (GraphPad Software, Inc., San Diego, CA, USA). The Mann–Whitney U test was used to analyze the percentage of fluorescent cells, flow cytometry findings, and qPCR results.

## Supplementary Information

Supplementary Information.
