# Supplementary material for: Transposase-CRISPR mediated targeted integration (TransCRISTI) in the human genome
Source: Sci Rep. 2022 Mar 1;12:3390. doi: 10.1038/s41598-022-07158-8 (PMC8888626; doi:10.1038/s41598-022-07158-8)
Supplement: Supplementary file 1 — Supplementary Information. [file 41598_2022_7158_MOESM1_ESM.docx]

**Transposase-CRISPR mediated targeted integration (TransCRISTI) in the human genome**

Mahere Rezazade Bazaz ^a, b^, Mohammad M. Ghahramani Seno ^a, c^,
Hesam Dehghani ^a, b, c, *^

*^a^ Division of Biotechnology, Faculty of Veterinary Medicine, Ferdowsi University of Mashhad, Mashhad, 9177948974, Iran.^b^ Stem Cell Biology and Regenerative Medicine Research Group, Research Institute of Biotechnology, Ferdowsi University of Mashhad, 9177948974, Mashhad, Iran.^c^ Department of Basic Sciences, Faculty of Veterinary Medicine, Ferdowsi University of Mashhad, Mashhad, 9177948974, Iran.*

* Corresponding author

Hesam Dehghani (ORCID ID: 0000-0001-6750-0040)
Address: Research Institute of Biotechnology, Ferdowsi University of Mashhad, Azadi Square, Mashhad, Iran. 9177943369. Tel: +98-51-3880-3795; Fax: +98-51-3880-7029. Email: [dehghani@um.ac.ir](mailto:dehghani@um.ac.ir)

# Supplementary Figures and Tables


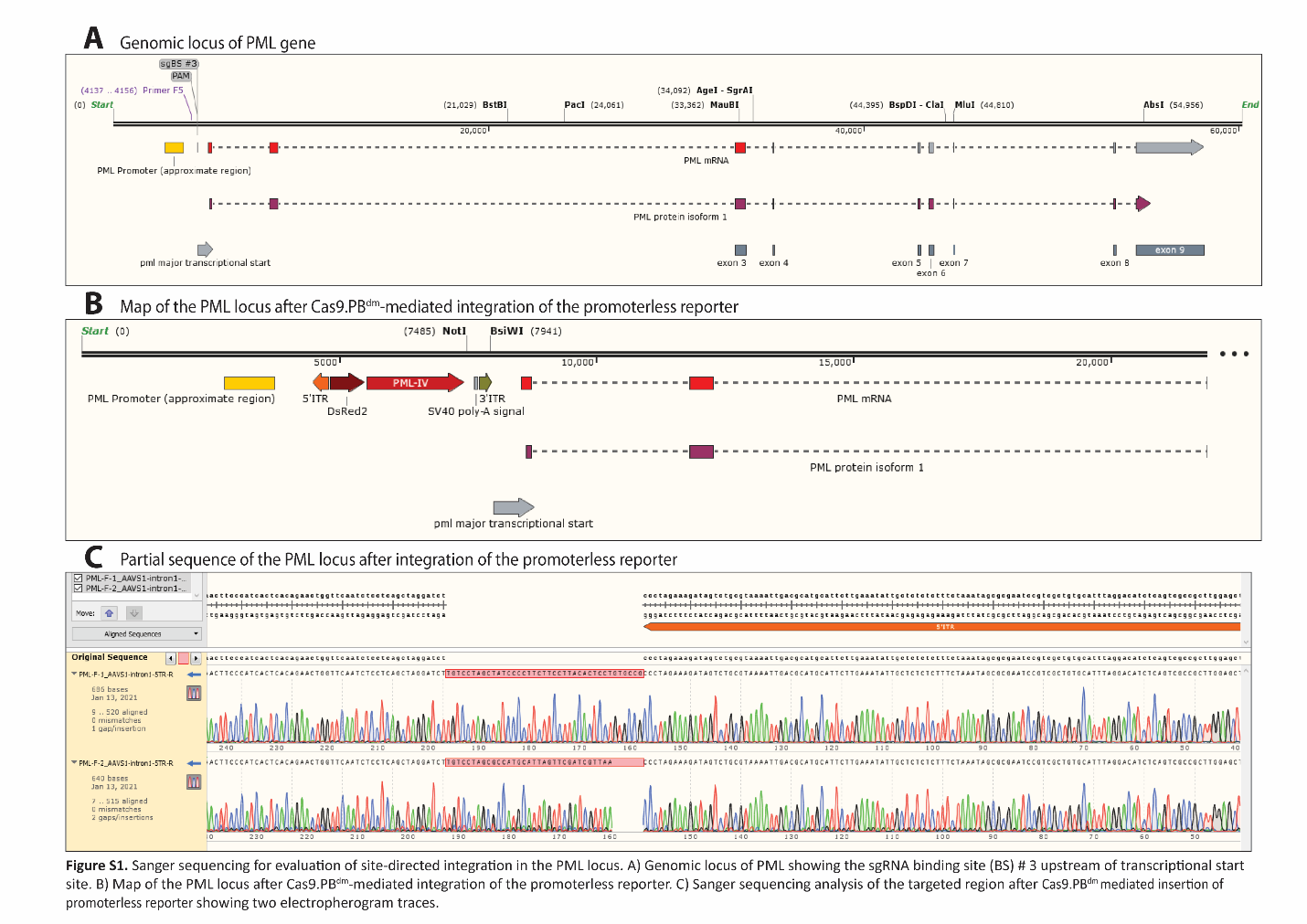


**Figure S1.** Sanger sequencing for evaluation of site-directed integration in the PML locus. A) Genomic locus of PML showing the sgRNA binding site (BS) # 3 upstream of transcriptional start site. B) Map of the PML locus after Cas9.PB^dm^ mediated integration of the promoterless reporter. C) Sanger sequencing analysis of the targeted region after Cas9.PB^dm^ mediated insertion of promoterless reporter showing two electropherogram traces.


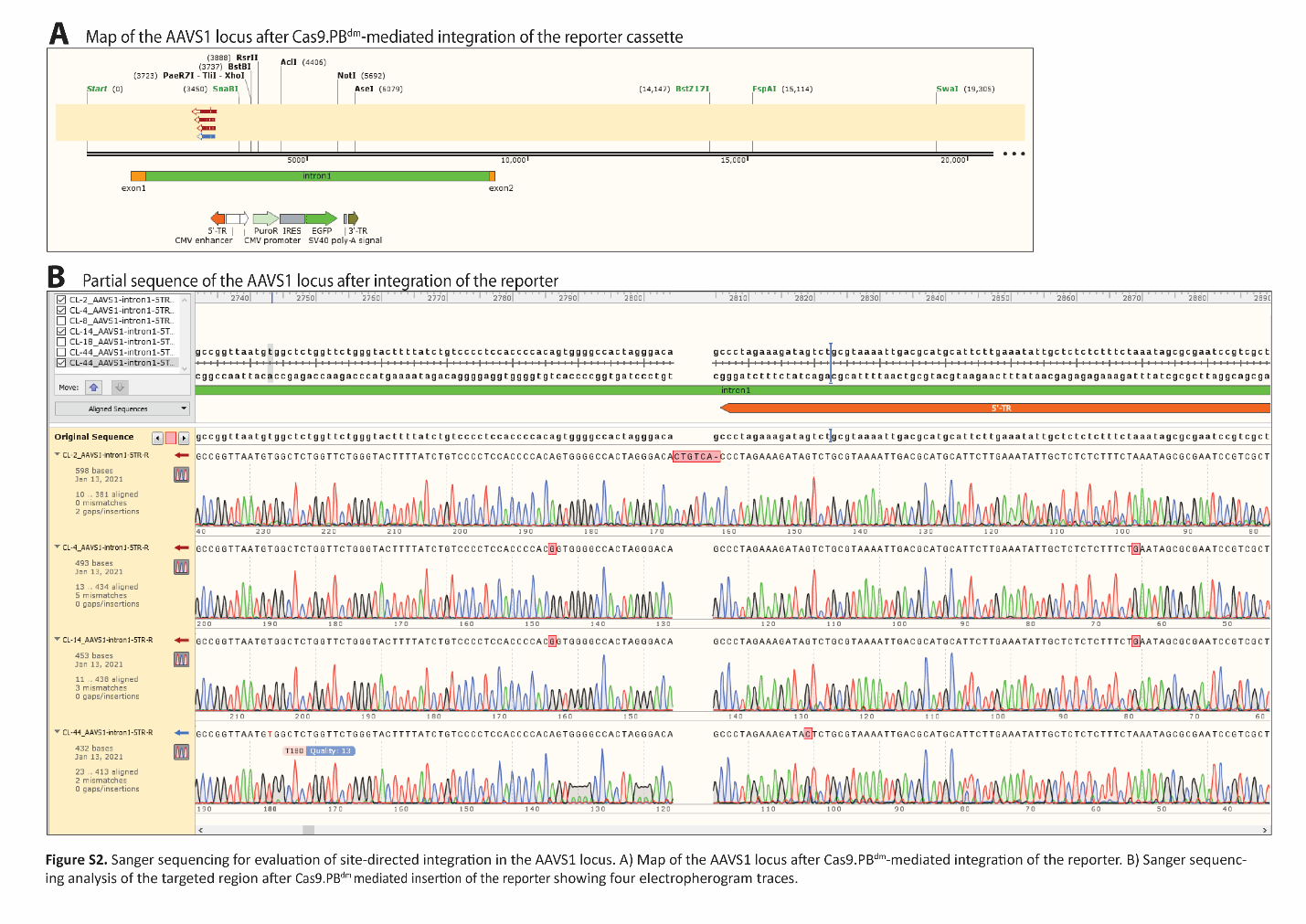


**Figure S2**. Sanger sequencing for evaluation of site-directed integration in the AAVS1 locus in different isogenic cell lines. A) Map of the AAVS1 locus after Cas9.PB^dm^ mediated integration of the reporter. B) Sanger sequencing analysis of the targeted region after Cas9.PB^dm^ mediated insertion of the reporter showing four electropherogram traces.

| Table S1. Plasmid constructs used in this study | | | |
| --- | --- | --- | --- |
| Plasmid | Features | Application | |
| a) Plasmids encoding effector enzymes/sgRNAs | | |  |
| pHX_3501 | CMV-PB-bGH PA | Encoding wild type *piggyBa*c transposase | |
| pHX_5001 | CAG-SV40 NLS-PB^dm^-bGH PA | Encoding mutant *piggyBac* transposase | |
| pHX_5002 | hU6-sgRNA #1-CAG-Cas9-SV40 NLS-linker-PB^dm^-bGH PA | Encoding sgRNA #1 for targeting pHDF_8001, and Cas9.PB^dm^ | |
| pHX_5004 | hU6-sgRNA #2-CAG-Cas9-SV40 NLS-linker-PB^dm^-bGH PA | Encoding sgRNA #2 for targeting the human AAVS1 gene locus, and Cas9.PB^dm^ | |
| pHX_5005 | hU6-sgRNA #3-CAG-Cas9-SV40 NLS-linker-PB^dm^-bGH PA | Encoding sgRNA #3 for targeting the human PML gene locus, and Cas9.PB^dm^ | |
| pHX_5009 | hU6-No sgRNA-CAG-Cas9-SV40 NLS-linker-PB^dm^-bGH PA | Encoding Cas9.PB^dm^ | |
| pHX_459 | hU6-No sgRNA-CAG-SV40 NLS-Cas9-T2A-PuroR-bGH PA | Encoding Cas9 | |
| pHX_4591 | hU6-sgRNA #3-CAG-SV40 NLS-Cas9-T2A-PuroR-bGH PA | Encoding sgRNA #3 for targeting the human PML gene locus, Cas9, and puromycin resistance gene | |
| pHX_4592 | hU6-sgRNA #1-CAG-SV40 NLS-Cas9-T2A-PuroR-bGH PA | Encoding sgRNA #1 for targeting pHDF_8001, Cas9, and puromycin resistance gene | |
| PHX_4593 | hU6-sgRNA #2-CAG-SV40 NLS-Cas9-T2A-PuroR-bGH PA | Encoding sgRNA #2 for targeting the human AAVS1 gene locus, and Cas9 | |
| b) Transposon/donor plasmids | | | |
| pHDS_502 | CMV-5’ITR-SA-T2A-EGFP-PA-3’ITR-mCherry-SV40 PA | Transposon cassette to assay the reversibility of cassette integration | |
| pHD_4012 | 5’ITR-CMV-PuroR-IRES-EGFP-SV40 PA-3’ITR | Transposon cassette encoding puromycin resistance gene and EGFP | |
| pHD_4015 | 5’ITR-CMVenhancer-3’ITR | Transposon cassette containing CMV enhancer | |
| pHDS_600 | 5’ITR-SA-T2A-EGFP-SV40 PA-3’ITR | Transposon cassette encoding EGFP | |
| pHDS_610 | 5’ITR-DsRed2-PML4-SV40 PA-3’ITR | Transposon cassette encoding DsRed2 fused to PML4 | |
| pHDS_612 | SgRNA #3 binding site (+PAM)-5’ITR-DsRed2-PML-SV40 PA-3’ITR | Transposon cassette encoding DsRed2-PML4 and providing binding site for sgRNA #3 | |
| pHDF_8001 | DsRed2-PML4-SV40 PA | Acceptor plasmid for interplasmid transposition assay | |
| pHD_40191 | 5’ITR-CMV-3’ITR | Donor plasmid for interplasmid transposition assay | |
| c) Plasmids used for bacterial expression of transposases | | | |
| pHD_77 | T7 promoter-Lac operator-pH.NpuC-PB-T7 terminator | Inducible expression of N-terminally-intein tagged wild type *piggyBac* | |
| pHD_78 | T7 promoter-Lac operator-pH.NpuC-PB^dm^-T7 terminator | Inducible expression of N-terminally-intein tagged mutant *piggyBac* | |
| bGH PA: bovine growth hormone polyadenylation signal  CAG: CAG promoter containing the cytomegalovirus early enhancer element, the first exon and the first intron of chicken beta-actin gene, and the splice acceptor of the rabbit beta-globin gene;  Cas9: SpCas9, CRISPR associated protein 9 from *Streptococcus pyogenes*  Cas9.PB^dm^: SpCas9 fused to double mutant mammalian codon-optimized *piggyBac* transposase CMV: CMV promoter containing human cytomegalovirus immediate early enhancer and promoter; DsRed2: red fluorescent protein (a mutant form of DsRed from Discosoma sp.) EGFP: enhanced green fluorescent protein hU6: human U6 promoter;  IRES: internal ribosome entry site;  ITR: *piggyBac* transposon terminal repeats; PB: mammalian codon-optimized *piggyBac* transposase;  PB^dm^: double mutant mammalian codon-optimized *piggyBac* transposase;  pH.NpuC: C-fragment of the split intein from Nostoc punciforme DnaE that exhibits enhanced pH sensitivity PML4: the gene encoding human promyelocytic leukemia isoform 4 PuroR: Puromycin resistance gene encoding puromycin N-acetyltransferase SA: splice acceptor sequence;  sgRNA: single guide RNA region encoding a custom-designed short crRNA (CRISPR RNA) sequence fused to the scaffold tracrRNA (trans-activating RNA) sequence;  SV40 NLS: SV40 nuclear localization sequence.  SV40 PA: SV40 polyadenylation signal sequence; T2A: self-cleaving peptide sequence; | | | |

| Table S2. Oligo sequences and their target locations | | |
| --- | --- | --- |
| Oligo | Sequence (5’ to 3’) | Target location |
| a) sgRNA sequences ^a^ | | |
| SgRNA #1 | F: cacccgccatgcattagttattac  R: aaacgtaataactaatgcatggcg | pHDF_8001 |
| SgRNA #2 | F: caccggggccactagggacaggat  R: aaacatcctgtccctagtggcccc | AAVS1 gene locus (ACNO. S51329) |
| SgRNA #3 | F: caccgtcttgtcctagcacactcct  R: aaacaggagtgtgctaggacaagac | PML gene locus (ACNO. X91752) |
| SgRNA #3 binding site (+PAM) | F: ggatcttgtcctagcacactcctat  R: taaggagtgtgctaggacaagatcc | PML gene locus (ACNO. X91752) & pHDS_612 (sgRNA BS #3 +PAM) |
| b) primer sequences for site directed mutagenesis | | |
| primer mPB-F1  primer mPB-R1 | F: gaataatga**gaattc^*^**gcctatccctatgacgtgcccgattatg  R: gatctctct***ggc***gttgct***ggc***cacggtgcc | Linker DNA between Cas9 and pBdm coding sequences  mPB (ACNO. EF587698.1) |
| primer mPB-F2  primer mPB-R2 | F: ggcaccgtg***gcc***agcaac***gcc***agagagatc  R: gaattatc**ctcgag^**^**cggccgctcatcagaaac | mPB (ACNO. EF587698.1)  mPB (ACNO. EF587698.1) |
| c) primer sequences for PCR recovery of insertion sites | | |
| primer 1 (F1)  primer 2 (R2) | F: ccgcgtgagtcaaaatgacg  R: ggtgcttcacgtacaccttg | pHD_40191, pHDF_8001 |
| primer 3 (F3)  primer 4 (R4) | F: ctcctgtggattcgggtcac  R: cgtcattttgactcacgcgg | AAVS1 gene locus (ACNO. S51329), pHDS_600, PHD_4012 |
| primer 5 (F5)  primer 6 (R6) | F: tccattggcgaagacctagc  R: cagactatctttctagggtta | PML gene locus (ACNO. X91752), pHDS_610 |
| primer 7 (F8) | F: aaagtgaagtgatctcctgctgc | Chr 17 (ACNO. AC005224) |
| primer 8 (F9) | F: ccacgttctcggtctccctg | Chr 5 (ACNO. AC110005) |
| primer 9 (F10) | F: ggccttgggacttagggcaa | Chr 11 (ACNO. AP000445) |
| primer 10 (F11) | F: tggaaaatgggctctgccaaca | Chr X (ACNO. NG_007488 ) |
| primer 11 (F12) | F: tgtggacagtggctatggtggt | Chr 16 (ACNO. AC130465) |
| primer 12 (R13) | R: gaagagttcttgcagctcgg | PHD_4012 |
| primer 13 (R14) | R: cgggccatttaccgtaag | PHD_4012 |
| primer 14 (F15) | R: gcggataacaatttcacacagg | PHD_4015 |
| primer 15 (R16) | R: gtaaaacgacggccagt | PHD_4015 |
| Primer 16 (R17) | R: ctctggctccatcgtaagca | AAVS1 gene locus (ACNO. S51329) |
| Primer 17 (F18) | F: tcatatgccaagtacgcccc | CMV promoter |
| Primer 18 (R19) | R: ccctcgatctcgaactcgtg | mCherry gene |
| d) Primer sequences for qPCR | | |
| primer 17 (F17)  primer 18 (R18) | F: aagctgaccctgaagttcatctgc  R: gatggtgcgctcctggac | EGFP gene |
| primer 19 (F19)  primer 20 (R20) | F: agatttggacctgcgagcg  R: gagcggctgtctccacaagt | RNase P gene |
| ^a^ F and R oligos generate overhangs upon annealing. These overhangs (shown as underlined sequences) are used for direct cloning of annealed oligos into the restriction enzyme sites of the target plasmid. The cacc and aacc overhangs (underlined) were used for cloning into the BbsI-linearized plasmid and at and ta overhangs were used for cloning into the PvuI-linearized plasmid.  mPB: Mouse codon optimized *piggyBac*. Bold underlined sequences denote the restriction enzyme recognition sequences; * EcoRI recognition site, ** XhoI recognition site. *Bold italic sequences* denote the mismatched alanine amino acid codon sequence which were introduced into the wild type mPB sequence to generate PB ^R372A/K375A^.  Chr: chromosome.  ACNO: NCBI accession number. | | |

| Table S3. The location of predicted insertion sites in the human genome and the amplification^1^ results on each insertion site, in cell lines derived from TransCRISTI and CRISPR-HITI knockin | | | |
| --- | --- | --- | --- |
| Potential off-target integration sites  (Chromosome No./ accession number) | TransCRISTI | CRISPR-HITI | |
| Chromosome 17/AC005224 | - | | - |
| Chromosome 5/AC110005 | - | | - |
| Chromosome 11q/AP000445 | - | | + |
| Chromosome X/NG_007488 | - | | - |
| Chromosome 16/AC130465 | - | | + |
| ^1^ Sequences of primers used for amplification are in Table S2. | | | |


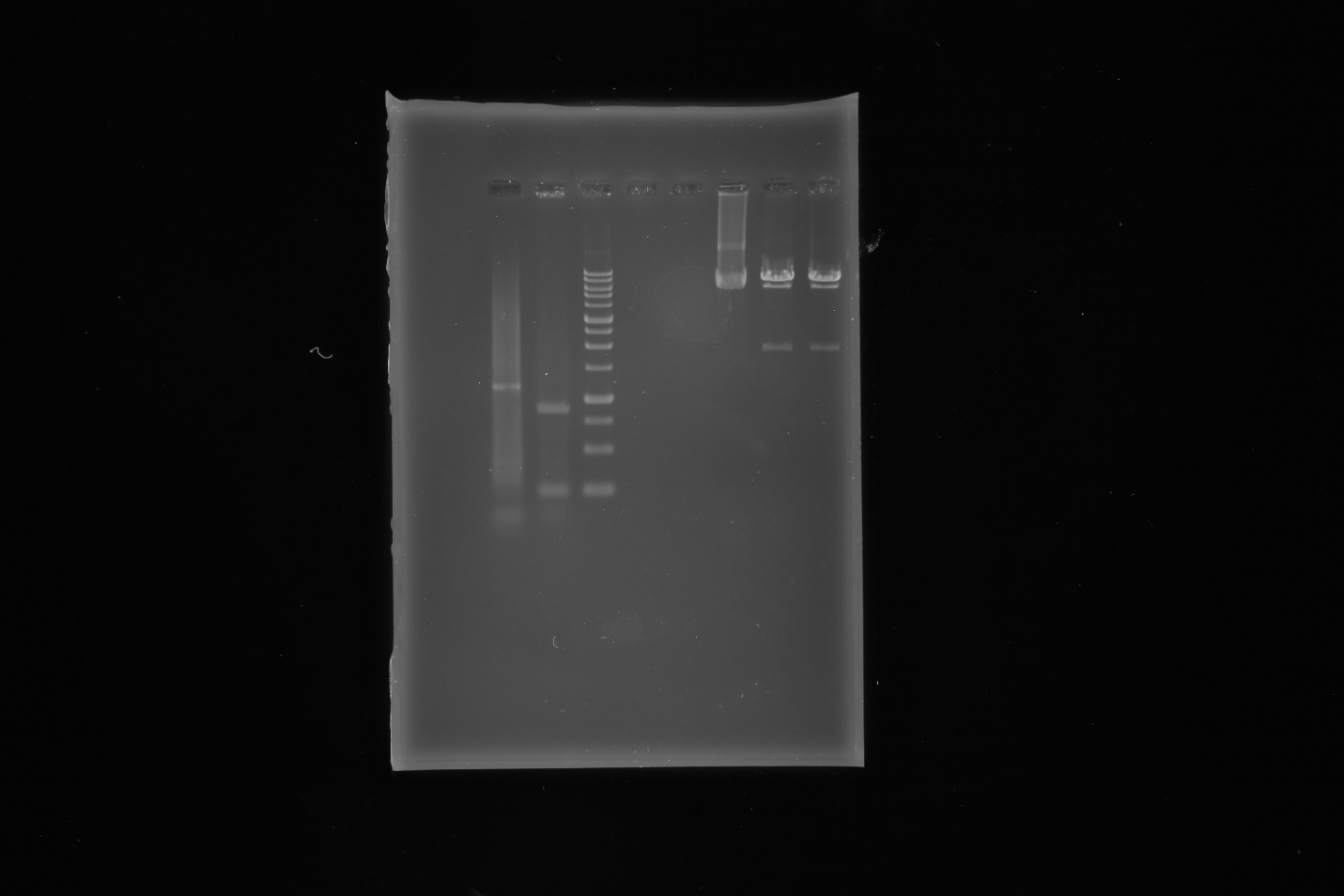


Image of the full-length gel used in Figure 1.


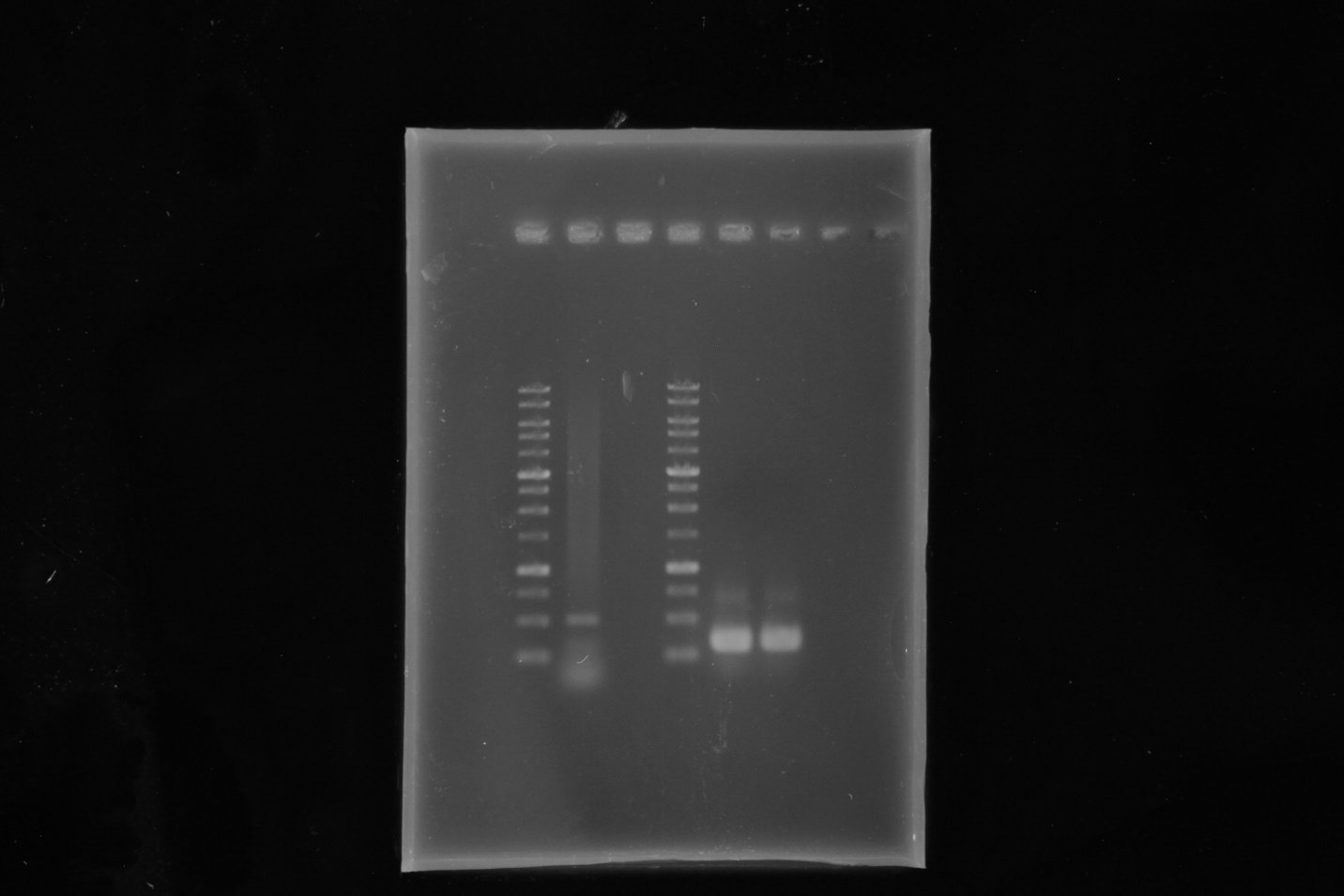


Image of the full-length gel used in Figure 2.


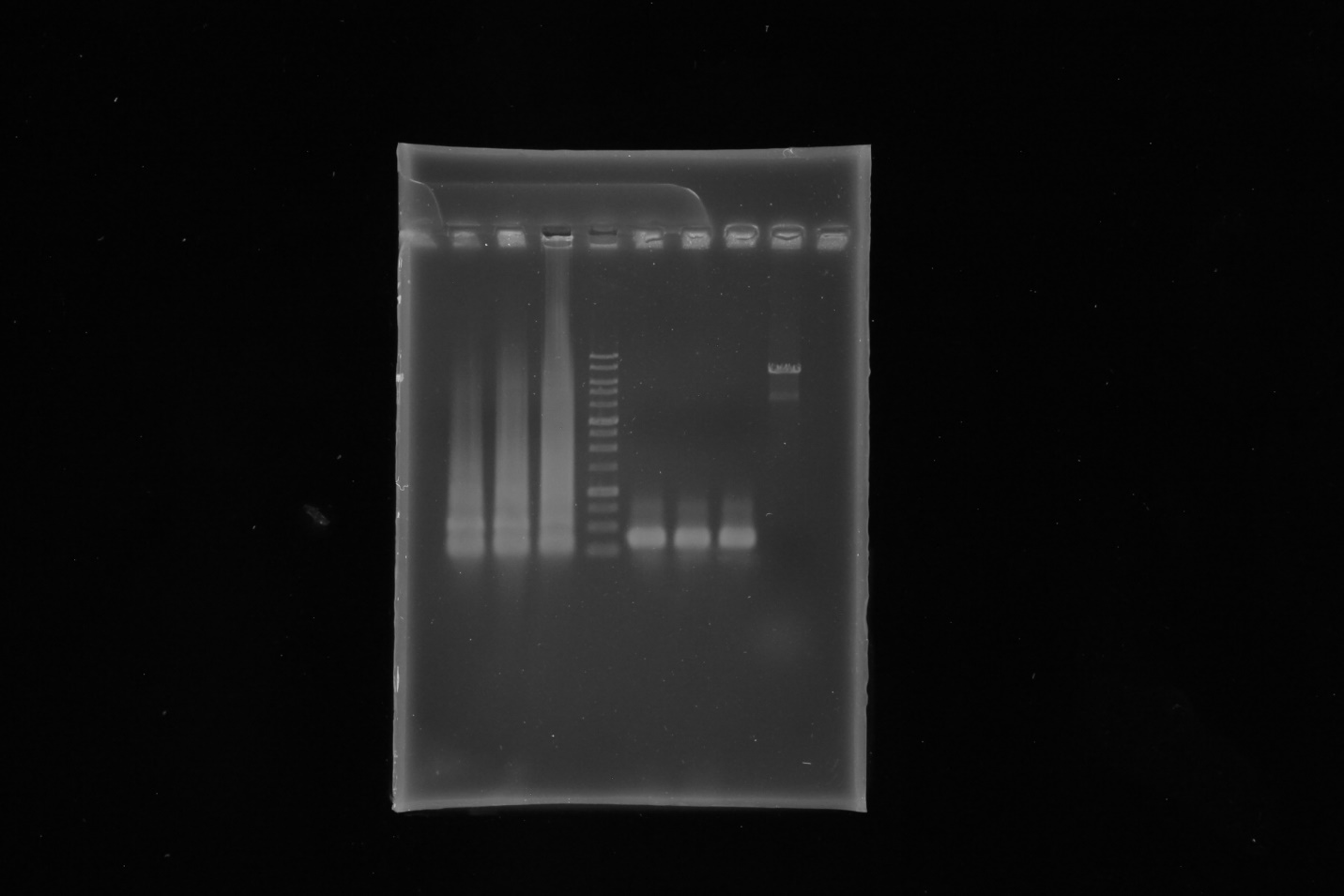


Image of the full-length gel used in Figure 3.


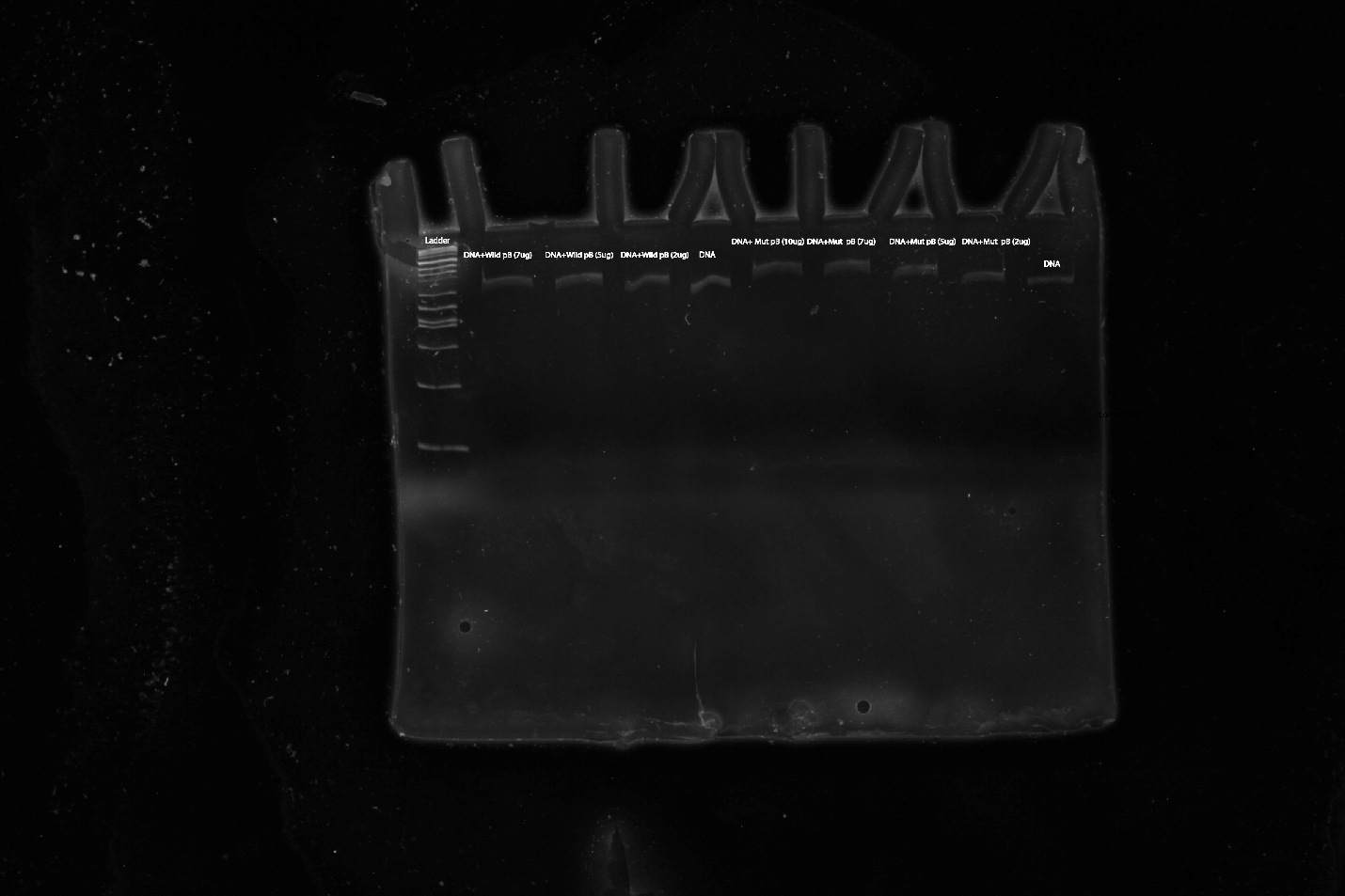


Image of the full-length gel used in Figure 4.
